# Amino acid deprivation triggers a novel GCN2-independent response leading to the transcriptional reactivation of non-native DNA sequences

**DOI:** 10.1371/journal.pone.0200783

**Published:** 2018-07-18

**Authors:** Annarosaria De Vito, Massimo Lazzaro, Ilaria Palmisano, Davide Cittaro, Michela Riba, Dejan Lazarevic, Makoto Bannai, Davide Gabellini, Maria Vittoria Schiaffino

**Affiliations:** 1 Division of Genetics and Cell Biology, IRCCS San Raffaele Scientific Institute, Milan, Italy; 2 Center for Translational Genomics and Bioinformatics, IRCCS San Raffaele Scientific Institute, Milan, Italy; 3 Frontier Research Labs, Institute for Innovation, Ajinomoto Co., Kawasaki, Tokyo, Japan; University of Wisconsin Madison, UNITED STATES

## Abstract

In a variety of species, reduced food intake, and in particular protein or amino acid (AA) restriction, extends lifespan and healthspan. However, the underlying epigenetic and/or transcriptional mechanisms are largely unknown, and dissection of specific pathways in cultured cells may contribute to filling this gap. We have previously shown that, in mammalian cells, deprivation of essential AAs (methionine/cysteine or tyrosine) leads to the transcriptional reactivation of integrated silenced transgenes, including plasmid and retroviral vectors and latent HIV-1 provirus, by a process involving epigenetic chromatic remodeling and histone acetylation. Here we show that the deprivation of methionine/cysteine also leads to the transcriptional upregulation of endogenous retroviruses, suggesting that essential AA starvation affects the expression not only of exogenous non-native DNA sequences, but also of endogenous anciently-integrated and silenced parasitic elements of the genome. Moreover, we show that the transgene reactivation response is highly conserved in different mammalian cell types, and it is reproducible with deprivation of most essential AAs. The General Control Non-derepressible 2 (GCN2) kinase and the downstream integrated stress response represent the best candidates mediating this process; however, by pharmacological approaches, RNA interference and genomic editing, we demonstrate that they are not implicated. Instead, the response requires MEK/ERK and/or JNK activity and is reproduced by ribosomal inhibitors, suggesting that it is triggered by a novel nutrient-sensing and signaling pathway, initiated by translational block at the ribosome, and independent of mTOR and GCN2. Overall, these findings point to a general transcriptional response to essential AA deprivation, which affects the expression of non-native genomic sequences, with relevant implications for the epigenetic/transcriptional effects of AA restriction in health and disease.

## Introduction

In animals, excessive, insufficient, or imbalanced nutrient availability is known to strongly impact on phenotype and health, both short and long-term, and across generations [1, 2]. In particular, studies in yeast, animal models and humans have shown that reduced food intake, reducing either overall calories, or only sugars, proteins, or even single amino acids (AA), such as Methionine (Met), may extend lifespan and healthspan, and reduce the risk of cancer and other age-related diseases [3–9]. In addition, fasting or specific AA deprivation have shown potential therapeutic applications, owing to their ability to directly reduce the growth of some tumor types [10, 11], sensitize cancer cells to chemo- or immunotherapy [12, 13], and allow efficient hematopoietic stem cell engraftment [14]. However, little is known about the specific processes and molecular mechanisms mediating the roles of nutrient restriction in human health and longevity.

A properly balanced diet in metazoans contains optimal amounts of a subset of AA, which cannot be synthetized *de novo* and are therefore named essential amino acids (EAAs). In humans these include Met, Histidine (His), Isoleucine (Ile), Leucine (Leu), Lysine (Lys), Phenylalanine (Phe), Threonine (Thr), Tryptophan (Trp), and Valine (Val), while a few others are considered as semi-essential, such as Glutamine (Gln) and Tyrosine (Tyr) [15, 16]. Consistently, EAA deprivation triggers a cell-autonomous adaptive response, characterized by extensive metabolic and gene expression modifications, implementing biosynthetic, catabolic, and plasma membrane transport processes, aimed at reconstituting the full AA complement [17, 18]. The best known and conserved pathways responding to AA deprivation are triggered by mechanistic Target of Rapamycin Complex 1 (mTORC1) and General amino acid Control Non-derepressible 2 (GCN2) protein kinases [15, 19, 20]. Activation of mTORC1 requires in particular the presence of Gln, Arg and Leu, but also Met [21], which activate the kinase through sensors mainly acting upstream of Rag GTPases at lysosomal membranes [22]. In turn, mTORC1 promotes cell growth, proliferation and anabolism upon activation, and translational attenuation and autophagy upon inhibition [19, 20].

By contrast, GCN2 is activated by deprivation of any individual EAA, by means of its histidyl-tRNA synthetase-related domain, which binds uncharged tRNAs accumulating during AA limitation [23, 24]. Upon activation, GCN2 phosphorylates and inhibits its only known downstream target, namely the eukaryotic Initiation Factor 2 α (eIF2α), thereby initiating the Integrated Stress Response (ISR). This leads to attenuation of general translation, and induction of a transcriptional/translational program, aimed at increasing stress resistance and restoring cell homeostasis, by upregulating a specific subset of genes, including Activating Transcription Factor 4 (ATF4) and C/EBP-Homologous Protein (CHOP) [25–27]. Thus, inhibition of mTORC1 and activation of GCN2 by AA restriction cooperate to attenuate general translation at the initiation step, increase catabolism and turnover, and enhance stress resistance to promote adaptation [15]. However, how these processes eventually induce protective mechanisms against the alterations associated with aging, which include pervasive epigenetic and transcriptional changes [28, 29], remains largely unknown.

We previously reported the unexpected observation that prolonged deprivation of either Tyr, or of both Methionine and Cysteine (Met/Cys), triggers the selective and reversible reactivation of exogenous transcriptional units, including plasmids, retroviral vectors and proviruses, integrated into the genome and transcriptionally repressed by defensive mechanisms against non-native DNA sequences [30, 31]. This phenomenon was observed both in HeLa epithelial and ACH-2 lymphocytic human cells, and was independent of the transgene or provirus (Ocular Albinism type 1, OA1; Green Fluorescent Protein, GFP; Lysosomal-Associated Membrane Protein 1, LAMP1; Human Immunodeficiency Virus-1, HIV-1), or of the exogenous promoter driving their transcription, either viral (cytomegalovirus, CMV; Long Terminal Repeat, LTR) or human (Phospho-Glycerate Kinase 1, PGK1; Elongation Factor-1α, EF-1α) [30]. Furthermore, this transgene reactivation response was not reproduced by serum starvation, activation of p38, or pharmacological inhibitors of mTOR (PP242 or rapamycin), sirtuins and DNA methylation. By contrast, it was induced by pan histone deacetylase (HDAC) inhibitors, and by selective inhibitors of class II HDACs [30]. Consistently, we found that the mechanism responsible involves epigenetic modifications at the transgene promoter, including reduced nucleosome occupancy and increased histone acetylation, and is mediated in part by reduced expression of a class II HDAC, namely HDAC4 [30].

These findings indicate that AA deprivation induces a specific epigenetic and transcriptional response, affecting the expression of newly-integrated exogenous transgenes and proviruses, and suggesting that endogenous sequences sharing similar structural and functional features may represent a transcriptional target as well [30, 31]. In particular, transposable elements, such as LTR-retrotransposons (or endogenous retroviruses, ERVs), are genomic “parasites” anciently-integrated into the genome, and silenced by epigenetic mechanisms of mammalian cells against the spreading of mobile elements, eventually becoming "endogenized" during evolution [32, 33]. This raises the question of whether their expression is also sensitive to AA restriction. In addition, it remains unclear whether or not the transgene reactivation response is related to specific AA deprivations, and most importantly which is the AA sensing/signaling pathway involved, in particular whether the GCN2 kinase is implicated. Thus, here we used the reactivation of silenced transgenes in cultured cells, as a model to investigate a novel molecular pathway induced by imbalanced EAA starvation, implicated in the epigenetic/transcriptional regulation of exogenous non-native DNA sequences and possibly of other endogenous anciently-integrated genomic elements.

## Materials and methods

### Cell lines and media

HeLa human epithelial carcinoma, HepG2 human hepatocellular carcinoma and C2C12 mouse skeletal muscle cells were maintained in DMEM containing glutaMAX (Invitrogen) and supplemented with 10% FBS (Sigma), 100 U/ml penicillin G (Invitrogen), 100 mg/ml streptomycin (Invitrogen), at 37°C in a 5% CO_2_ humidified atmosphere. Cell lines carrying integrated and partially silenced transgenes were also maintained in 600–1000 μg/ml G418.

### Cell line authentication

The C2C12 cell line was provided by ATCC. HeLa and HepG2 cells were obtained by Drs. F. Blasi and G. Tonon at San Raffaele Scientific Institute, Milan, Italy, respectively, and were authenticated by Short Tandem Repeat (STR) profiling, using the Cell ID System kit (Promega), according to the manufacturer’s instructions. Briefly, STR-based multiplex PCR was carried out in a final volume of 25 μL/reaction, including 5 μL Cell ID Enzyme Mix 5X, 2.5 μL Cell ID Primer Mix 10X and 3 ng of template DNA. The thermal cycling conditions were: 1 cycle at 96°C for 2 min, followed by 32 cycles at 94°C for 30 sec, 62°C for 90 sec, and 72°C for 90 sec, and 1 cycle at 60°C for 45 sec. The following STR loci were amplified: AMEL, CSF1PO, D13S317, D16S539, D21S11, D5S818, D7S820, TH01, TPOX, vWA. Fragment length analysis of STR-PCR products was performed by Eurofins Genomics, using standard procedures of capillary electrophoresis on the Applied Biosystems 3130 XL sequencing machine, and assessment of the STR profile was performed at the online STR matching analysis service provided at http://www.dsmz.de/fp/cgi-bin/str.html.

### Expression vectors and stable transfection

Stable cell clones, expressing myc-tagged human OA1 (GPR143) or GFP transcripts, were generated using pcDNA3.1/OA1myc-His or pcDNA3.1/EGFP vectors [30]. Briefly, HeLa, HepG2 and C2C12 cells were transfected using FuGENE 6 (Roche) and selected with 800, 1000, and 650 μg/ml of G418 (Sigma), respectively, which was maintained thereafter to avoid loss of plasmid integration. G418-resistant clones were isolated and analyzed for protein expression by epifluorescence and/or immunoblotting.

### AA starvation

Full DMEM-based medium, carrying the entire AA complement, and media deprived of Met/Cys (both AAs), Met (only), Cys (only), Alanine (Ala), Thr, Gln, Val, Leu, Tyr, Trp, Lys and His were prepared using the Nutrition free DMEM (cat.#09077–05, from Nacalai Tesque, Inc., Kyoto, Japan), by adding Glucose, NaHCO_3_, and either all 20 AAs (for full medium) or 18–19 AAs only (for deprivations of two-one AAs). Single AAs, Glucose, and NaHCO_3_ were from Sigma. Further details and amounts utilized are indicated in **S1 Table**. All media were supplemented with 10% dialyzed FBS (Invitrogen), 100 U/ml penicillin G (Invitrogen), 100 mg/ml streptomycin (Invitrogen), and G418 as required. HBSS was from Invitrogen. Cells were seeded at 10–30% of confluency; cells to be starved for 48 h were plated 2–3 times more confluent compared to the control. The following day, cells were washed and cultured in the appropriate medium, with or without EAA, for 24–48 h.

### Reagents and cell treatments

L-Histidinol (HisOH), PP242, Integrated Stress Response Inhibitor (ISRIB), SP600125, Cycloheximide (CHX) were from Sigma; Salubrinal was from Tocris Bioscience; U0126 was from Promega. Drugs were used at the following final concentrations: HisOH at 4–16 mM; PP242 at 1–3 μM; ISRIB at 100 nM; SP600125 at 20 μM in HepG2 cells and 50 μM in HeLa cells; Cycloheximide (CHX) at 50 ug/ml in HepG2 cells and 100 ug/ml in HeLa cells; Salubrinal at 75 μM; U0126 at 50 μM. Vehicle was used as mock control. Treatments with drugs to be tested for their ability to inhibit transgene reactivation (ISRIB, SP600125 and U0126) were initiated 1h before the subsequent addition of L-Histidinol (ISRIB) or the subsequent depletion of Met/Cys (SP600125 and U0126).

### RNA extraction, cDNA synthesis and real-time PCR

Total RNA was purified using the RNeasy Mini kit (Qiagen), according to manufacturer’s instructions. RNA concentration was determined by Nanodrop 8000 Spectrophotometer (Thermo Scientific). Equal amount (1 μg) of RNA from HeLa, HepG2 and C2C12 cells was reverse transcribed using the SuperScript First-Strand Synthesis System for RT-PCR (Invitrogen) using oligo-dT as primers, and diluted to 5 ng/μl. The cDNA (2 μl) was amplified by real-time PCR using SYBR green Master Mix on a Light Cycler 480 (Roche), according to manufacturer’s instructions. The thermal cycling conditions were: 1 cycle at 95°C for 5 min, followed by 40–45 cycles at 95° for 20 sec, 56° for 20 sec and 72° for 20 sec. The sequences, efficiencies and annealing temperatures of the primers are provided in **S2 Table**. Data were analyzed with Microsoft Excel using the formula E_target_^Δct target (control-sample)^ /E_reference_^Δct reference (control-sample)^ [34]. Reference genes for normalizations were ARPC2 (actin-related protein 2/3 complex, subunit 2) for HeLa and HepG2 cells; and Actb (actin beta) for C2C12 cells, unless otherwise indicated.

### RNAi and CRISPR/Cas9 genomic editing

siRNA (Mission esiRNA, 200 ng/μL; Sigma) against ATF4 and GCN2 were designed against the targeted sequences NM_001675 and NM_001013703, respectively. Cells seeded in 6-well plates were transfected with 1 μg of siRNAs and 5 μL of Lipofectamine 2000 (Invitrogen), following manufacturer’s instructions, at day 1 post-plating for ATF4 and at day 1 and 2 post-plating for GCN2. At day 2 (ATF4) or 3 (GCN2) post-plating, cells were washed and cultured in medium in the absence or presence of HisOH 4 mM for 6 h. siRNAs against RLuc (Sigma), targeting Renilla Luciferase, were used as negative control. For CRISPR/Cas9 experiments, we used the “all-in-one Cas9-reporter” vector, expressing GFP (Sigma), which is characterized by a single vector format including the Cas9 protein expression cassette and gRNA (guide RNA). GFP is co-expressed from the same mRNA as the Cas9 protein, enabling tracking of transfection efficiency and enrichment of transfected cells by fluorescence activated cell sorting (FACS). The human U6 promoter drives gRNA expression, and the CMV promoter drives Cas9 and GFP expression. The oligonucleotide sequences for the three gRNAs targeting GCN2 exon 1 or 6 are listed in **S2 Table.** We transfected HeLa and HepG2 cells with these plasmids individually (one plasmid one guide) and sorted the GFP-positive, transfected cells by FACS. Screening GCN2-KO clones was performed by western blotting. In the case of HepG2-OA1 cells, two rounds of selection were necessary to obtain three GCN2-KO clones by using a guide RNA against exon 1. Compared to the original HepG2-OA1 cell line and to the clone resulting from the first round of selection (185#27), the selected clones E23, F22 and F27 showed a very low amount—if any—of residual GCN2 protein (see results).

### DNA extraction and conventional PCR amplification

Genomic DNA of HeLa and HepG2 cells was purified using DNeasy Blood and Tissue kit (Qiagen), according to the manufacturer’s instructions. DNA concentration was determined by Nanodrop 8000 Spectrophotometer (Thermo Scientific). PCR conditions for amplification of GCN2 exon 1 and 6 were as follows: 1 cycle at 94°C for 5 min, followed by 35 cycles at 94°C for 40 sec, 56°C for 40 sec, and 72°C for 40 sec; and a final extension step of 5 min at 72°C. The primer sequences are provided in **S2 Table**.

### Western immunoblotting

For OA1, western immunoblotting was carried out as described [35]. For GCN2, cells were lysed in RIPA buffer, boiled at 95°C for 5 min and resolved on a 7.5% polyacrylamide gel; immunoblotting was then performed following standard procedures. Primary Abs were as follows: anti-human OA1, previously developed by our group in rabbits [35]; anti-GCN2 (Cell Signaling, Cat. #3302).

### Statistical analysis

Statistical analyses were performed using Microsoft Excel for Mac (version 15.32, Microsoft) for Student’s t-test; or GraphPad Prism (version 5.0d for Mac, GraphPad Software, Inc.) for one-way analysis of variance (ANOVA), followed by Dunnett’s or Tukey’s multiple comparisons post-tests. T-test was used when only two means, typically sample versus control, were compared, as specified in the figure legends. One way ANOVA was used for multiple comparisons, followed by either a Dunnett’s (to compare every mean to a control mean), or a Tukey’s (to compare every mean with every other mean) post-test, by setting the significance level at 0.05 (95% confidence intervals). Both tests compare the difference between means to the amount of scatter, quantified using information from all the groups. Specifically, Prism computes the Tukey-Kramer test, allowing unequal sample sizes. P values in Figures are generally referred to comparison between a sample and the control (full medium/mock), and are indicated as follows: *P<0.05, **P<0.01, ***P<0.001. Comparisons not involving the control are similarly indicated, by a horizontal line at the top of the graphs, encompassing the two samples under analysis. Additional details regarding the specific experiments are reported in the Figure Legends.

### Transcriptome analysis of HeLa cells

To examine the expression behavior of genomic repeats upon AA starvation, we performed a transcriptomic analysis taking advantage of an intramural sequencing facility. HeLa-OA1 cells were cultured in normal medium (for 6-30-120 hours) or in absence of Met/Cys (for 6-15-30-72-120 hours). Total RNA was prepared using Trizol (Sigma) to preserve transcripts of both small and long sizes (from Alu, of about 0.3 kb, to Long Interspersed Nuclear Elements, LINEs, and ERVs, up to 6–8 kb long), DNase treated to avoid contamination of genomic DNA, and processed for NGS sequencing by Ovation RNA-Seq System V2 protocol and HiSeq 2000 apparatus. Raw sequence data (10–20 M reads/sample) were aligned to the human genome (build hg19) with SOAPSplice [36]. Read counts over repeated regions, defined by RepeatMasker track from UCSC genome browser [37], were obtained using bedtools suite [38]. Normalization factors and read dispersion (d) were estimated with edgeR [39], variation of abundance during time was analyzed using maSigPro package [40], fitting with a negative binomial distribution (Θ = 1/d, Q = 0.01), with a cutoff on stepwise regression fit r^2^ = 0.7. Read counts were transformed to RPKM for visualization purposes. The expression of the OA1 transgene and HDAC4, which are progressively up- and down-regulated during starvation, respectively [30], were used as internal controls.

For genomic repeat analysis, reads belonging to repetitive elements were classified according to RepeatMasker and assigned to repeat classes (total number in the genome = 21), families (total number in the genome = 56) and finally subfamilies (total number in the genome = 1396), each including a variable number of genomic loci (from a few hundred for endogenous retroviruses, up to several thousand for Alu). Repeat subfamilies were then clustered according to their expression pattern in starved vs control cells, by maSigPro using default parameters, and repeats classes or families that are significantly enriched in each cluster, compared to all genomic repeats, were identified by applying a Fisher Exact test (using scipy.stats, a statistical module of Python). Alternatively, differentially expressed repeat subfamilies were identified by averaging three time points of starvation (15-30-72 h) and controls. Repeats significantly up- or downregulated (104 and 77, respectively) were selected based on a P value <0.05 (unpaired two-tailed Student’s t-test, assuming equal variance), and analyzed for their class enrichment by a Fisher Exact test as described above.

For gene set enrichment analysis of Met/Cys deprived vs control HeLa cells, differentially expressed genes were selected considering three time points of starvation (15-30-72 h) and controls, based on a P value <0.05 (unpaired two-tailed Student’s t-test, assuming equal variance) and a fold change >2. This led to a total of 2033 differentially expressed genes, 996 upregulated and 1037 downregulated. The enrichment analysis was performed separately for up and down regulated genes, or with all differentially expressed genes together (both), using the KEGG database. The analysis was performed with correction for the background of all expressed genes (about 13600 genes showing an average expression over 3 starvation and 3 control samples of at least 5 counts) and by using default parameters (adjusted P value and q-value cut-off of <0.05 and 0.2, respectively). Differentially expressed genes were also selected considering all starvation time points, as with genomic repeats, by maSigPro using default parameters, and a fold change of at least 1.5, leading to similar enrichment results (not shown). RNAseq gene expression data are available in the ArrayExpress database under the accession number E-MTAB-6452.

## Results

### EAA starvation induces the expression of endogenous retroviruses

**Fig 1:**
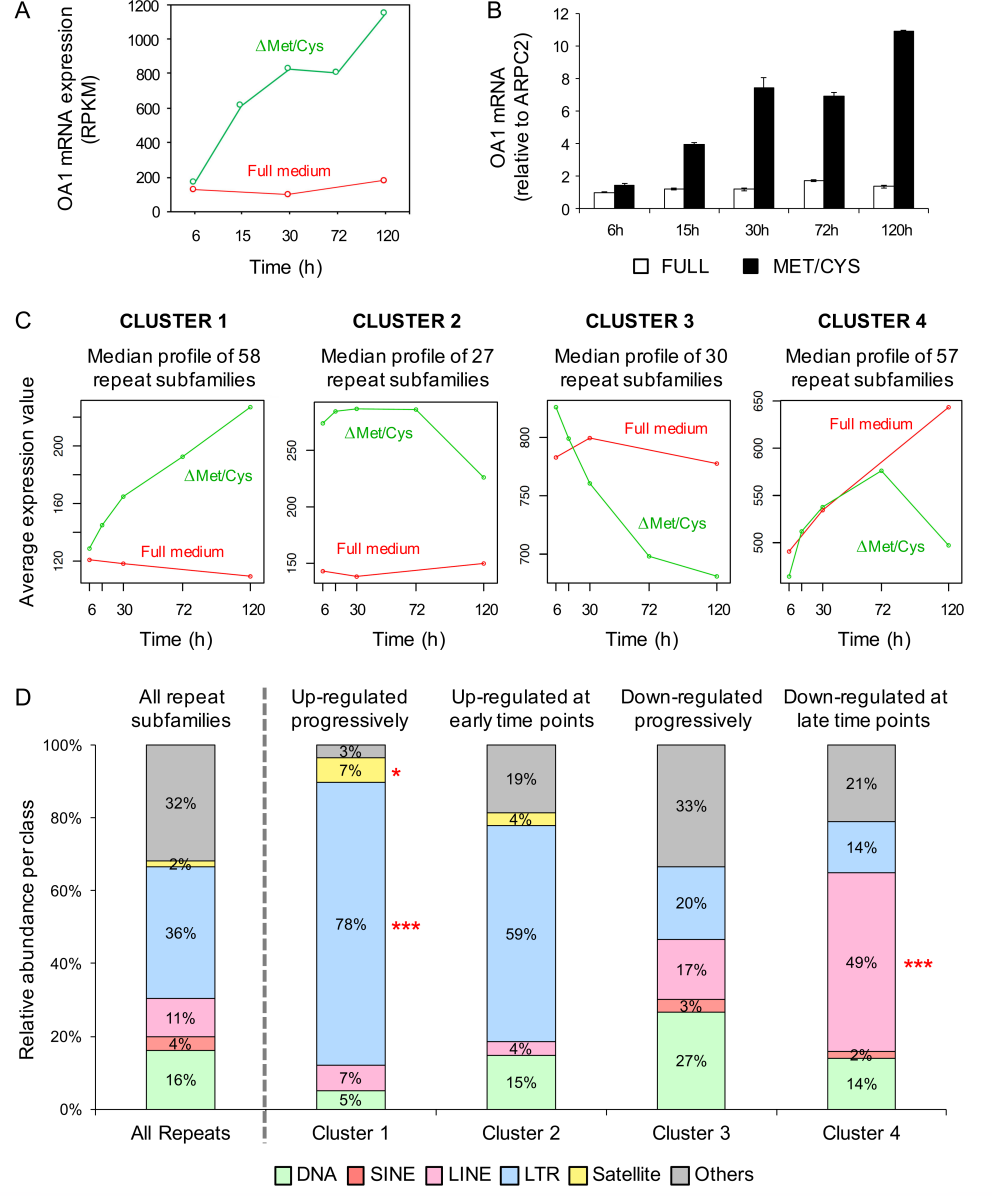
Exogenous transgene and endogenous retroviruses are upregulated in Met/Cys-deprived HeLa cells. (A,B) Exogenous integrated transgene (OA1) mRNA abundance in HeLa-OA1 cells, cultured in Met/Cys-deprived medium for the indicated time points, and analyzed by RNAseq (A), or RT-qPCR (B), compared to full medium. Data represent RPKM (A), or mean ± SD of 2 technical replicates, expressed as fold change vs. control (full medium at 6 h = 1) (B). (C) Clustering of 172 genomic repeat subfamilies, differentially expressed upon starvation, according to their expression profile. (D) Class distribution of repeat subfamilies belonging to differential expression clusters, compared to all genomic repeat subfamilies (first column). Class DNA includes DNA transposons; SINE includes Alu; LINE includes L1 an L2; LTR includes endogenous retroviruses and solitary LTRs; Satellite includes centromeric acrosomal and telomeric satellites; Others includes SVA, simple repeats, snRNA, and tRNAs. LTR-retroelements are significantly enriched among repeats that are upregulated upon starvation, while LINEs are significantly enriched among repeats that are downregulated. *P<0.05, ***P<0.001 (Fisher exact test).

To provide proof-of-principle that AA starvation may affect the expression of transposable elements, we performed an RNAseq analysis of the previously described HeLa-OA1 cells, carrying an integrated and partially silenced OA1 transgene [30]. Since the reactivation of the transgene by starvation is a progressive phenomenon [30], we performed a time-course experiment, where each time point represents one biological sample, rather than a biological triplicate of a single time point. To this aim, cells were cultured either in normal medium, or in absence of Met/Cys for different time points (6-15-30-72-120 hours), resulting in the progressive upregulation of the OA1 transgene during starvation (**Fig 1A and 1B**), consistent with previously published results [30]. The expression of genomic repeats was determined according to RepeatMasker annotation and classification into classes, families, and subfamilies. Repeat species were then subjected to differential expression and enrichment analyses in starved vs control conditions. Out of 1396 annotated repeat subfamilies, 172 species displayed a differential expression profile during starvation.

As shown in **Fig 1C**, the clustering of differentially expressed repeats, according to their expression pattern, reveals profiles comparable to the behavior of the transgene in the same conditions, i.e. upregulation upon starvation and no change in regular medium (Cluster 1 and 2). In particular, Cluster 1 contains sequences that, similarly to the OA1 transgene, are progressively upregulated upon starvation (**Fig 1A and 1C)** [30], while Cluster 2 contains sequences that are upregulated at early time points. Interestingly, repeat families that are significantly enriched in these two clusters belong mostly to the group of LTR-retrotransposons, including ERV1, ERVK, ERVL, ERVL-MaLR and other LTR sequences (**Fig 1D**; **S1A and S2A Figs**). By contrast, DNA transposons (such as TcMar-Tigger) and L1 non-LTR retrotransposons are enriched among repeats that are downregulated during starvation, particularly at late time points (Clusters 3 and 4) (**Fig 1D**; **S1A and S2A Figs**). Consistent results were obtained by selecting significantly up- or downregulated genomic repeats (overall 181 species), based on their average expression out of three time points of starvation (15-30-72 h, when the transgene upregulation is more homogeneous) and controls, and on a P value <0.05 (**S1B and S2B Figs**). These findings suggest that EAA starvation induces genome-wide effects involving repetitive elements, and that—among major repeat classes—it upregulates in particular the expression of ERVs.

**Fig 2:**
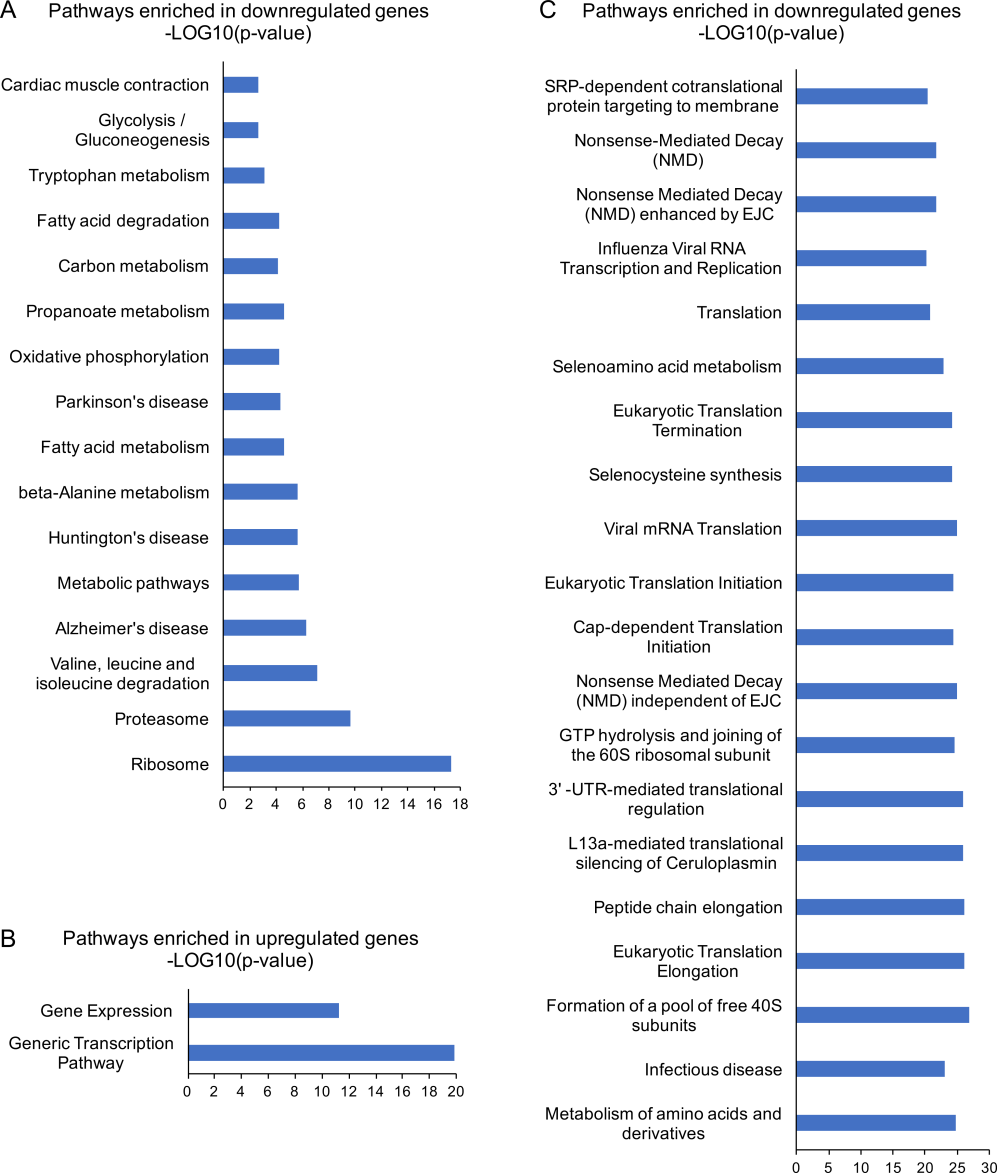
Gene set enrichment analysis of Met/Cys-deprived HeLa cells. Differentially expressed genes between three time points of starvation (15-30-72 h) and controls were selected based on a P value <0.05 and a fold change of at least 2, leading to a total of 996 upregulated, and 1037 downregulated genes. The enrichment analysis was performed separately for up and down regulated genes, using the EnrichR tool and the KEGG (A) and REACTOME (B, C) databases. Ranking is based on the combined score provided by EnrichR, and categories are displayed up to 20 items with an Adjusted P value <0.05. No significant categories were found with upregulated genes against the KEGG database. All data are shown in **S1 File**. The enrichment analysis using all differentially expressed genes together did not reveal any additional enriched process.

In addition, to obtain a general overview of main gene pathways changing their expression together with the transgene during AA starvation, we performed gene expression and enrichment analyses of regular genes, by considering three time points of starvation (15-30-72 h) and controls. Differentially expressed genes were selected based on a P value <0.05 and a fold change between means of at least 2, and analyzed with the EnrichR tool [41]. As shown in **Fig 2 and S1 File**, enrichment analyses against the KEGG and Reactome databases reveals a predominance of downregulated pathways, namely ribosome and translation, proteasome, AA metabolism, oxidative phosphorylation and other pathways related to mitochondrial functions, which are affected in Huntington, Alzheimer and Parkinson diseases (http://www.genome.jp/kegg/pathway.html). In particular, a large fraction of ribosomal protein mRNAs is downregulated upon Met/Cys starvation **(Fig 2A and 2C; S1 File),** consistent with the notion that their genes–despite being scattered throughout the genome—are coordinately expressed in a variety of conditions [42]. This reduced expression may depend on multiple pathways that control ribosome biogenesis in response to external stimuli, including the downregulation of Myc activity [43], the downregulation of mTORC1 [42, 44], or possibly the activation of the ISR, as described in yeast [45]. By contrast, upregulated genes show a significant enrichment for transcription and gene expression (**Fig 2B)**. Similar results were obtained by the Gene Ontology Biological Process (GO-BP) database **(S1 File)**, overall indicating a general downregulation of translation and metabolism, and upregulation of transcription, during the time interval of Met/Cys starvation corresponding to the transgene upregulation.

### The transgene reactivation response to EAA starvation is conserved in mammalian cells

To characterize the pathway leading to the reactivation of silenced transgenes, we used HeLa-OA1 and HeLa-GFP cells, as described [30]. In addition, to test cell types relevant for AA metabolism, such as liver and muscle, we generated clones of HepG2 human hepatoma and C2C12 mouse skeletal muscle cells, stably transfected with plasmids for OA1 and GFP transgenes, respectively (HepG2-OA1 and C2C12-GFP cells; endogenous OA1 is not expressed in any of these cell types). In all cases, the integrated transgenes are under the control of the CMV promoter in the context of a pcDNA3.1 plasmid, are partially silenced, and can be efficiently upregulated by HDAC inhibitors (trichostatin A, TSA; ref. [30] and **S3A, S3B and S4A Figs**), indicating that their expression is controlled at least in part by epigenetic mechanisms, as previously described [30].

**Fig 3:**
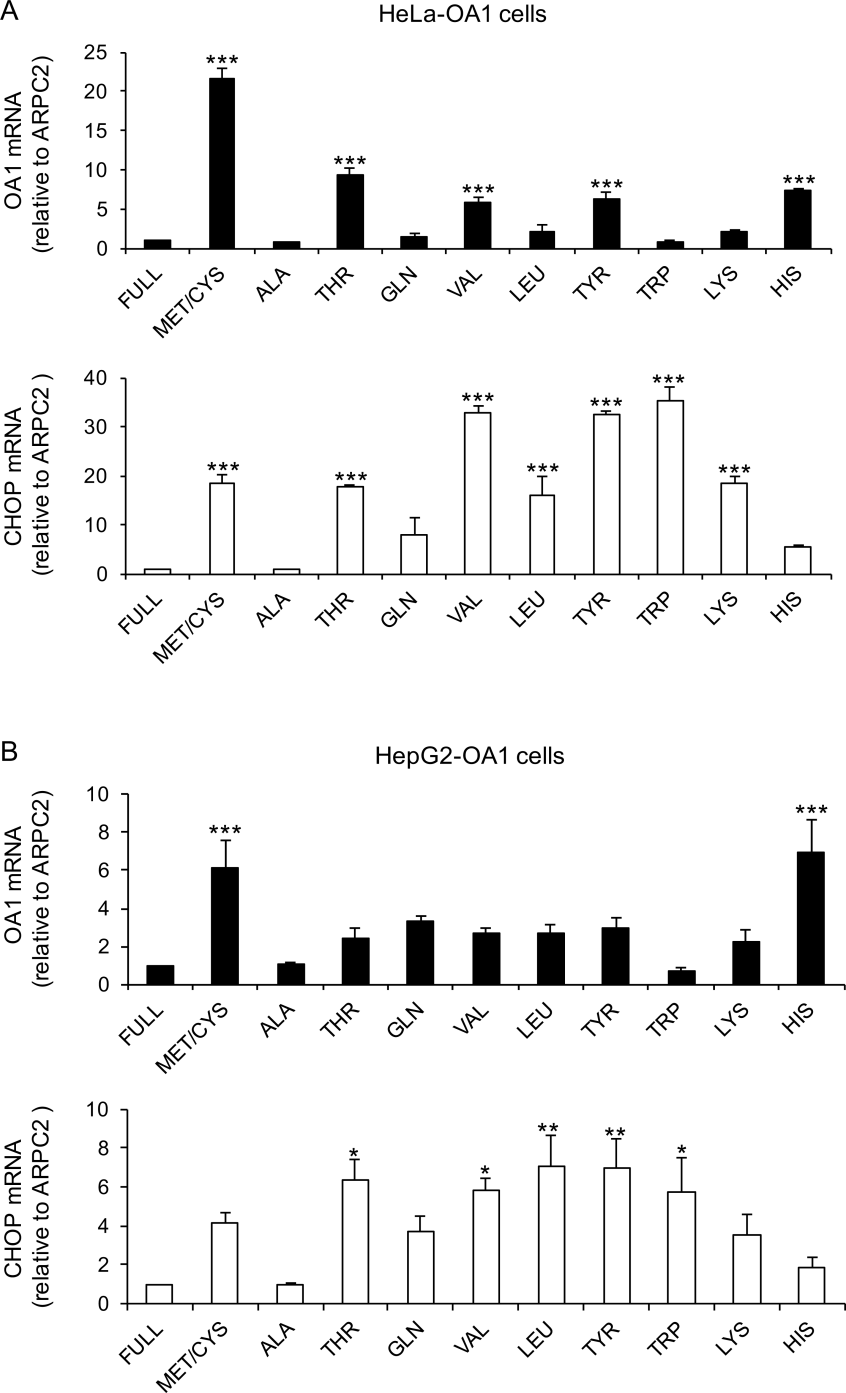
EAA deprivation induces reactivation of silent transgenes in HeLa and HepG2 cells. Relative transgene (OA1) and CHOP mRNA abundance in HeLa-OA1 (A) and HepG2-OA1 (B) cells, cultured in various AA-deprived media for 48 h and 24 h, respectively, compared to full medium. Mean ± SEM of 3 independent experiments. Data are expressed as fold change vs. control (full medium = 1). *P<0.05, **P<0.01, ***P<0.001 (one way ANOVA, followed by Dunnett’s post-test vs. full medium).

To establish whether the reactivation response results from the shortage of specific AAs only, such as Met/Cys, or it is triggered by any AA deprivations, we cultured HeLa-OA1, HeLa-GFP, HepG2-OA1 and C2C12-GFP cells for 24–48 hours with a battery of media deprived of EAAs or semi-EAAs, including Met/Cys, Thr, Gln, Val, Leu, Tyr, Trp, Lys, and His. As negative controls, cells were cultured in full medium, carrying the entire AA complement, and in a medium deprived of Ala, a non-essential AA. The expression of the transgene transcript was then evaluated by RT-qPCR. As shown in **Fig 3,** and in **S3C and S4B Figs**, most EAA-deficiencies induced reactivation of the OA1 or GFP transgenes in all four cell lines, with the notable exception of Trp deprivation, which consistently resulted in no or minimal reactivation of the transgenes. Indeed, despite some variability, Met/Cys deficiency, but also Thr, Val, Tyr, and His deprivation always gave an efficient response, while Leu, Gln and Lys elicited evident responses in some cases, but not in others. Depletion of Phe gave results comparable to Tyr deprivation, however it significantly altered multiple reference genes used for normalization and therefore was eventually omitted from the analysis (not shown). Finally, in the above experiments we used a combined Met/Cys deficiency, to avoid the potential sparing of Met by Cys [46] and for consistency with our previous studies [30]. Nevertheless, the analysis of single Met or Cys starvation, both at the protein and transcript levels, revealed an exclusive role of Met deprivation in transgene reactivation, consistent with the notion that Cys is not an EAA (**S3D and S3E Fig**).

Collectively, these results indicate that transgene reactivation by EAA starvation is reproducible with most EAAs, shared by different cell types (epithelium, liver, and skeletal muscle), and conserved in different mammalian species (human, mouse).

### The ISR is activated during prolonged EAA starvation

mTORC1 inhibition and GCN2 activation trigger the best-known signaling pathways responding to AA starvation [15]. We previously showed that inhibition of mTORC1 is not sufficient to reproduce transgene reactivation in HeLa cells [30]. By contrast, the involvement of GCN2 and the ISR, including the downstream effectors ATF4 and CHOP, has never been tested. In addition, this pathway has been typically assessed in transient assays, lasting for a few hours, which may not be comparable with the prolonged starvation conditions necessary to reactivate the transgene expression (at least 15–24 h). Thus, we tested whether CHOP expression was upregulated upon incubation of HeLa-OA1, HepG2-OA1 and C2C12-GFP cells in media deprived of different EAAs for 24–48 h.

As shown in **Fig 3** and **S4B Fig**, we found that CHOP expression is increased in all EAA-starvation conditions, but not in the absence of Ala, in all tested cell lines. Similar, yet less pronounced, results were obtained with ATF4, consistent with the notion that activation of this transcription factor is mainly mediated by translational upregulation (not shown) [15, 26]. However, the upregulation of CHOP does not parallel quantitatively that of the transgene, neither appears sufficient to induce it. In fact, CHOP is highly upregulated even upon Trp starvation, which consistently results in no or minimal reactivation of the transgenes (compare CHOP with OA1 or GFP expression; **Fig 3** and **S4B Fig**). Thus, while the ISR appears widely activated upon EAA starvation, the upregulation of its downstream effector CHOP only partly correlates with transgene reactivation and may not be sufficient to induce it.

### Pharmacological activators of GCN2 induce transgene reactivation similarly to starvation

**Fig 4:**
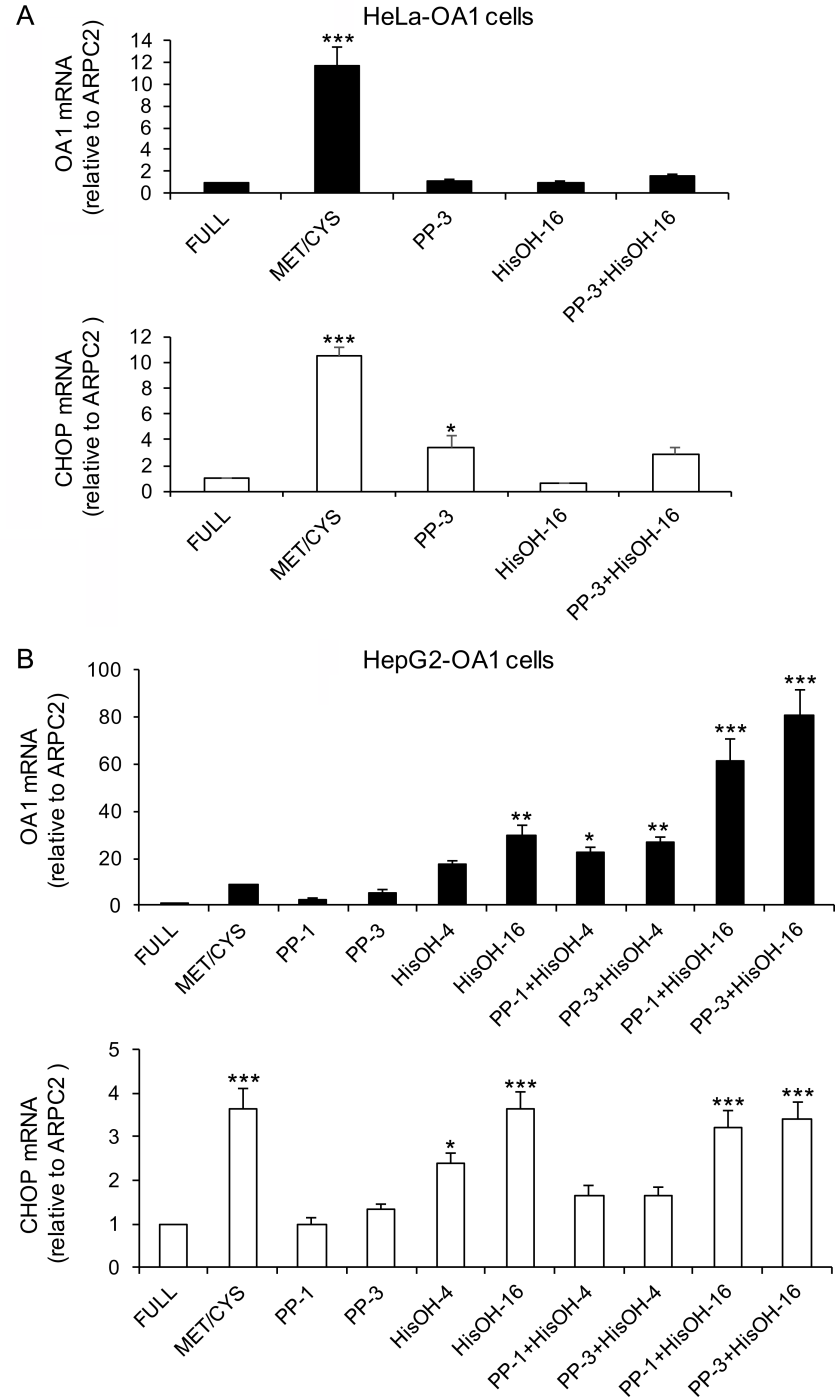
mTOR inhibition and GCN2 activation differently affect transgene expression in HeLa and HepG2 cells. Relative transgene (OA1) and CHOP mRNA abundance in HeLa-OA1 (A) and HepG2-OA1 (B) cells, cultured in Met/Cys-deprived medium, or in the presence of PP242 (mTOR inhibitor; 1–3 μM) or L-Histidinol (HisOH, GCN2 activator; 4–16 mM), either alone or in combination for 24–48 h, compared to full medium. Mean ± SEM of 4 (A) or 3 (B) independent experiments. Data are expressed as fold change vs. control (full medium = 1). *P<0.05, **P<0.01, ***P<0.001 (one way ANOVA, followed by Dunnett’s post-test vs. full medium). PP-1 and PP-3, PP242 at 1 and 3 μM, respectively; HisOH-4 and HisOH-16, L-Histidinol at 4 and 16 mM, respectively.

The activation of the ISR upon AA starvation suggests that GCN2 may be involved in the transgene reactivation response. Therefore, we tested whether direct pharmacological activation of this kinase is sufficient to trigger the transgene reactivation similarly to starvation. In addition, we used pharmacological inhibitors of mTOR to corroborate previous negative results in HeLa cells [30] in the other cell lines under study. To this aim, HeLa-OA1 or GFP, HepG2-OA1 and C2C12-GFP cells were cultured in the presence of different concentrations of PP242 (mTOR inhibitor) or L-Histidinol (GCN2 activator, inhibiting tRNA^His^ charging by histidyl-tRNA synthetase), either alone or in combination for 24 h, compared to Met/Cys-deprived and full medium. As shown in **Fig 4** and **S5 Fig**, while inhibition of mTORC1 consistently leads to minor or no effects, in agreement with previous findings [30], treatment with L-Histidinol results in efficient reactivation of the transgene in HepG2-OA1 and C2C12-GFP cells, but not in HeLa cells.

Specifically, L-Histidinol is not effective in HeLa-OA1 and HeLa-GFP cells, either alone or in combination with PP242 (**Fig 4A** and **S5A Fig**), or by using different concentrations of the drug, with or without serum (not shown). In these cells, L-Histidinol appears also unable to trigger the ISR, as indicated by lack of CHOP upregulation, possibly due to their different sensitivity to the drug. These findings are consistent with previous reports, describing the use of L-Histidinol in HeLa cells in conditions of low His concentration in the culture medium [47], which would resemble AA starvation in our system and therefore may not be applicable. Thus, even though the amount of the amino alcohol was adapted to exceed 20 to 80 times that of the amino acid, as described [47], HeLa cells may be resistant or able to compensate.

In contrast, in other cell types, L-Histidinol has been utilized in regular DMEM, to mimic the AA response triggered by DMEM lacking His [48, 49]. Consistently, in HepG2-OA1 cells, L-Histidinol is sufficient to elicit extremely high levels of transgene reactivation, and its combination with PP242 results in additive or even synergistic effects, possibly due to an indirect effect of mTOR inhibition on GCN2 activity (**Fig 4B**) [50, 51]. Similarly, C2C12-GFP cells efficiently reactivate the transgene upon treatment with L-Histidinol, but not PP242 (**S5B Fig**). However, differently from HepG2-OA1 cells, simultaneous treatment of C2C12-GFP cells with L-Histidinol and PP242 does not lead to synergistic effects. Consistent with stimulation of the ISR, CHOP and to a minor extent ATF4 are upregulated by L-Histidinol in both cell lines, yet their expression levels show only an incomplete correlation with those of the transgene (**Fig 4B**, **S5B Fig**, and not shown).

### The ISR is not implicated in the induction of transgene reactivation

**Fig 5:**
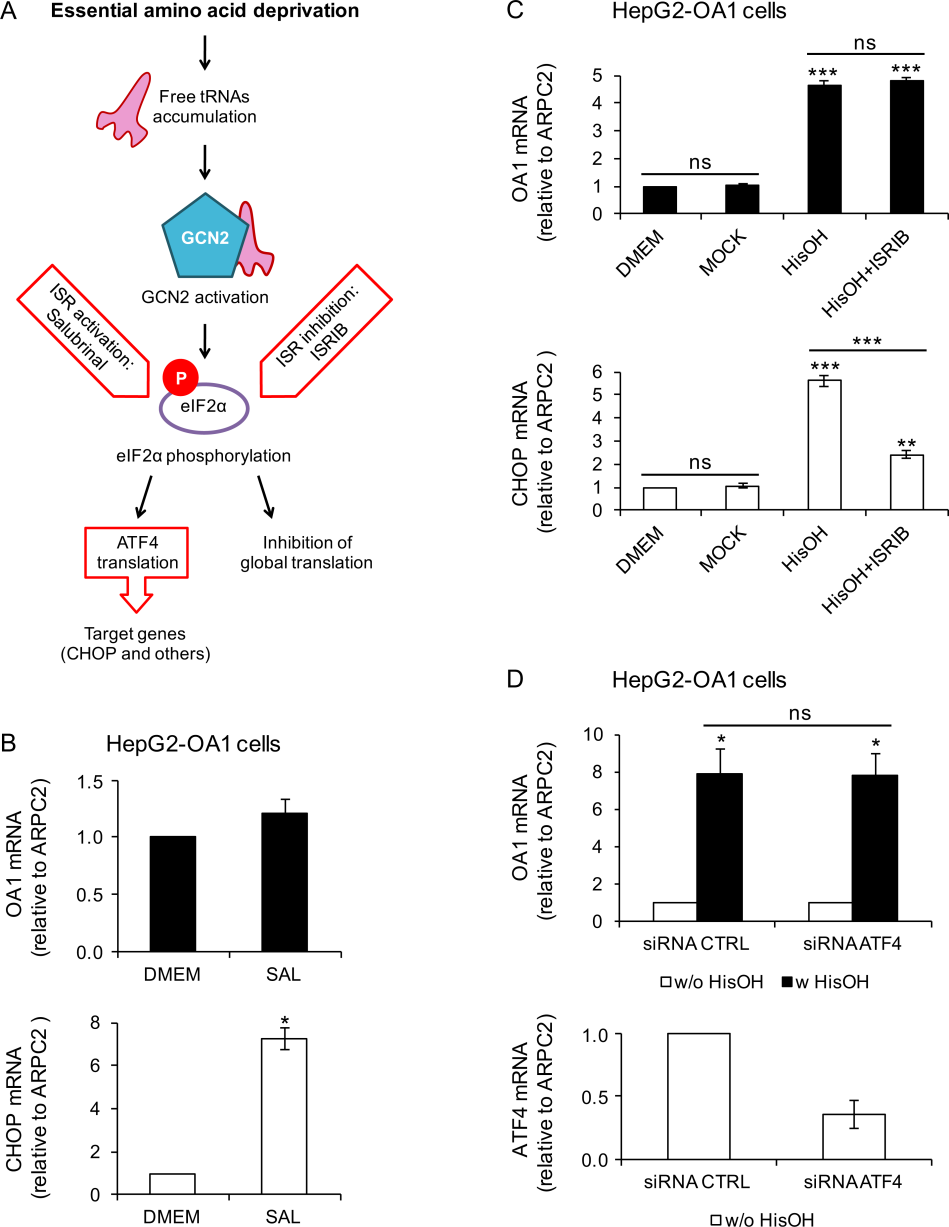
The ISR is neither sufficient nor necessary to induce transgene reactivation in HepG2 cells. (A) Schematic representation of GCN2 activation by AA starvation, resulting in phosphorylation of eIF2a and initiation of the downstream ISR. In addition to GCN2, the ISR may be activated by other eIF2a kinases (PKR, HRI and PERK; not shown in the picture). (B) Relative transgene (OA1) and CHOP mRNA abundance in HepG2-OA1 cells treated for 24 h with Salubrinal (a drug that induces the ISR by inhibiting the dephosphorylation of eIF2α; 75 μM), compared to full medium. Mean ± range of two experiments. Data are expressed as fold change vs. control (DMEM = 1). *P<0.05 (paired two-tailed Student’s t-test vs. control). (C) Relative transgene (OA1) and CHOP mRNA abundance in HepG2-OA1 cells treated for 6 h with L-Histidinol (HisOH, GCN2 activator; 4 mM), in the absence or presence of ISRIB (a drug that bypasses the phosphorylation of eIF2α, inhibiting triggering of the ISR; 100 nM). Mean ± range of two experiments. Data are expressed as fold change vs. control (DMEM = 1). **P<0.01, ***P<0.001 (one way ANOVA, followed by Tukey’s post-test; P values refer to comparisons vs. control, unless otherwise indicated). (D) Relative transgene (OA1) and ATF4 mRNA abundance in HepG2-OA1 cells transfected with control (CTRL) or anti-ATF4 siRNAs, and incubated in the presence or absence of L-Histidinol (HisOH, GCN2 activator; 4 mM) for 6 h. Mean ± range of two experiments. Data are expressed as fold change vs. control (w/o HisOH = 1, top; control siRNA = 1, bottom). *P<0.05 (one way ANOVA, followed by Tukey’s post-test; P values refer to comparisons vs. control, unless otherwise indicated).

The finding that GCN2 activation by L-Histidinol is sufficient to reactivate the transgenes in both HepG2-OA1 and C2C12-GFP cells pointed to this kinase, and to the downstream ISR, as the pathway possibly involved in the EAA starvation response. Thus, we investigated whether the ISR is sufficient to trigger upregulation of the OA1 transgene in HepG2-OA1 cells by pharmacological means. As CHOP expression does not correspond quantitatively and is not sufficient to induce transgene reactivation, we tested the role of the core upstream event of the ISR, namely the phosphorylation of eIF2α [26], which can be induced by pharmacological treatments, independent of GCN2 (**Fig 5A**). To this aim, we used Salubrinal, a specific phosphatase inhibitor that blocks both constitutive and ER stress-induced phosphatase complexes against eIF2α, thereby increasing its phosphorylation [52]. We found that, while the ISR is activated upon Salubrinal treatment, as shown by increased CHOP expression, it does not induce OA1 transgene reactivation (**Fig 5B**).

To test whether the ISR is necessary to trigger the transgene response to L-Histidinol, we used the chemical compound ISRIB, which inhibits the activation of the ISR, even in the presence of phosphorylated eIF2α, likely by boosting the activity of the guanine-nucleotide exchange factor (GEF) for eIF2α, namely eIF2B [53, 54]. HepG2-OA1 cells were stimulated with L-Histidinol, either in the presence or absence of ISRIB. As shown in **Fig 5C**, while the expression of CHOP is inhibited by ISRIB, as expected, the reactivation of the OA1 transgene is not affected. In addition, knockdown of the closest eIF2α downstream effector ATF4 by siRNAs does not interfere with the reactivation of the OA1 transgene by L-Histidinol (**Fig 5D**). Together, these data suggest that eIF2α phosphorylation and the downstream ISR pathway are neither sufficient nor necessary to induce transgene reactivation.

### GCN2 is not required for the transgene reactivation response to EAA starvation

**Fig 6:**
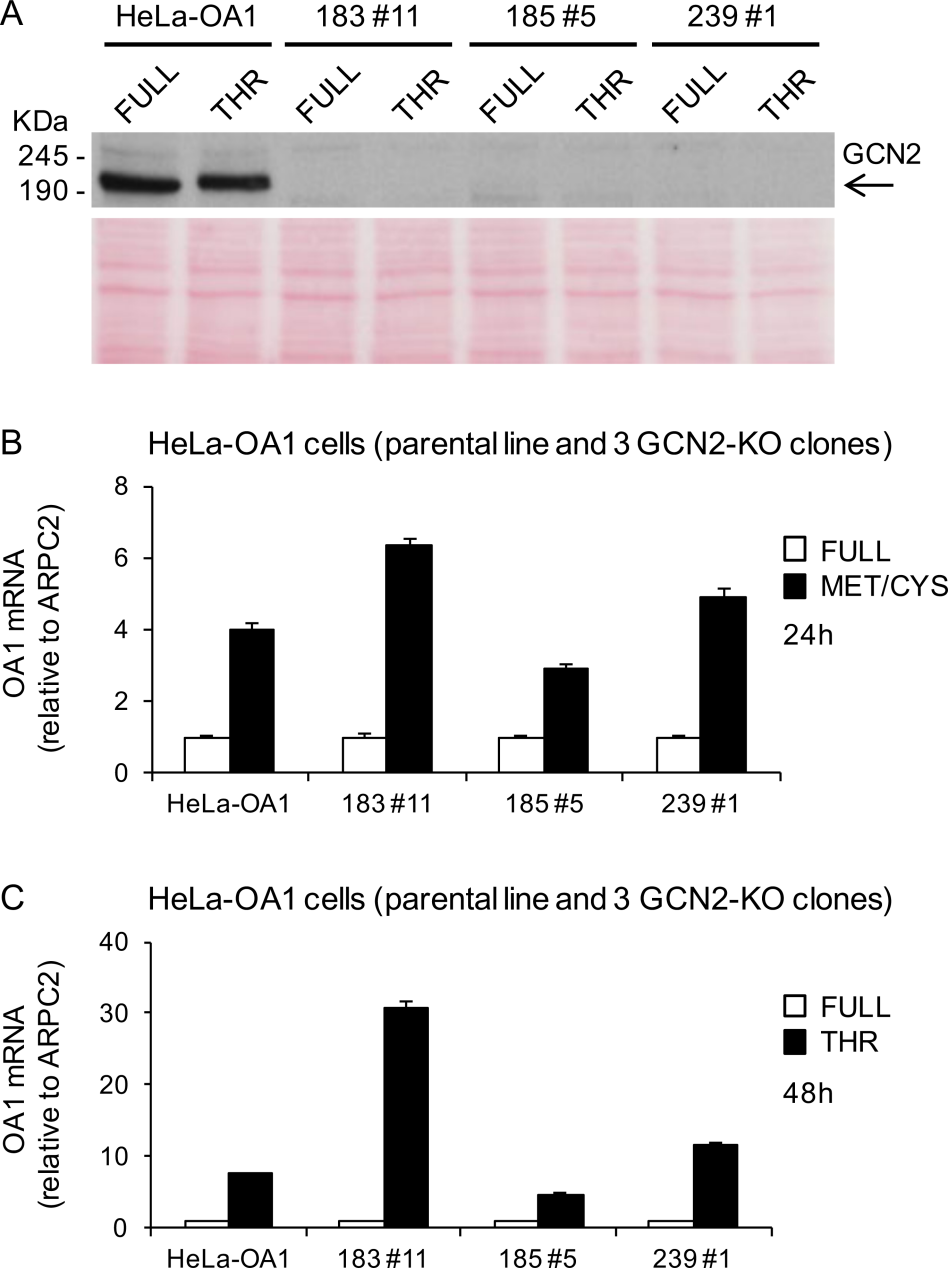
GCN2 knockout does not interfere with transgene reactivation in HeLa cells. (A) Immunoblotting of protein extracts from the HeLa-OA1 parental cell line and GCN2-KO clones 183#11, 185#5 and 239#1, immunodecorated with anti-GCN2 antibody. Arrow, GCN2 specific band. Ponceau staining was used as loading control. (B, C) Relative transgene (OA1) mRNA abundance in HeLa-OA1 cells and GCN2-KO clones, cultured in Met/Cys (B) or Thr (C) deprived medium for 24 h or 48 h, respectively, compared to full medium. Mean ± SD of 3 technical replicates from 1 experiment. Data are expressed as fold change vs. control (full medium = 1). Since independent clones may display variable reactivation responses (e.g. due to different levels of transgene expression in basal conditions), the results are not shown as means of the three clones, but as separate replicates.

To definitively establish if GCN2 is necessary to trigger the transgene reactivation response to EAA starvation, we directly suppressed its expression by CRISPR/Cas9-mediated knock-out (KO). We generated three independent GCN2-KO clones from the parental HeLa-OA1 cell line, by using three different guide RNAs, two against exon 1 (clones 183#11 and 185#5), and one against exon 6 (clone 239#1) of the GCN2 gene. Genomic characterization confirmed the presence of mutations on both alleles of exon 1 of the GCN2 gene in clone 183#11, and on both alleles of exon 6 in clone 239#1; by contrast, clone 185#5 showed multiple alleles in exon 1, consistent with the presence of two cell populations, and was not characterized further at the genomic level (**S6 Fig**). None of these clones express GCN2 at the protein level, as shown by immunoblotting (**Fig 6A**). To test the GCN2-KO cells for their ability to respond to EAA starvation, parental HeLa-OA1 cells and the three GCN2-KO clones were cultured in media deprived of Met/Cys or Thr (corresponding to the most effective treatments in this cell line; see **Fig 3A**) for 24–48 h and transgene expression was assessed by RT-qPCR. We found that the reactivation of the OA1 transgene is neither abolished, nor reduced by KO of GCN2, thus excluding that this kinase is necessary for the response to EAA starvation in HeLa-OA1 cells (**Fig 6B and 6C**).

**Fig 7:**
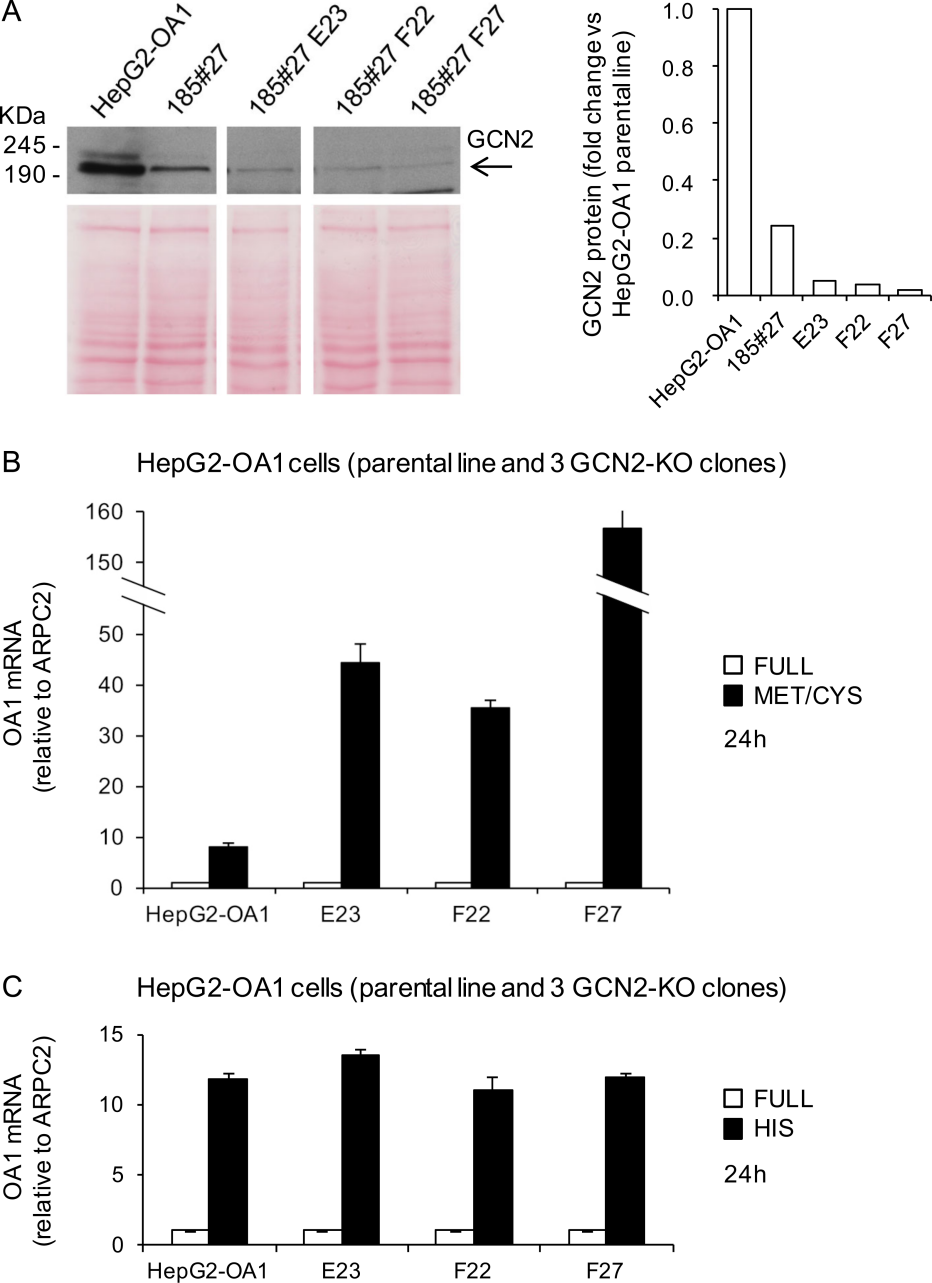
GCN2 knockout does not interfere with transgene reactivation in HepG2 cells. (A) Immunoblotting of protein extracts from the HepG2-OA1 parental cell line and GCN2-KO clones 185#27, E23, F22, F27, immunodecorated with anti-GCN2 antibody. Clone 185#27 results from the first round of selection, and was used to generate clones E23, F22, F27. Arrow, GCN2 specific band. For GCN2 protein quantification, Ponceau staining was used as loading control and data are expressed as fold change vs. parental cell line (= 1). (B, C) Relative transgene (OA1) mRNA abundance in HepG2-OA1 cells and GCN2-KO clones, cultured in Met/Cys (B) or His (C) deprived medium for 24 h, compared to full medium. Mean ± SD of 3 technical replicates from 1 experiment. Data are expressed as fold change vs. control (full medium = 1). Since independent clones may display variable reactivation responses (e.g. due to different levels of transgene expression in basal conditions), the results are not shown as means of the three clones, but as separate replicates. The greater reactivation of the transgene in the HepG2 clones (particularly upon Met/Cys deprivation) depends on the lower expression level of the transgene in basal conditions, probably secondary to subculturing, resulting in a higher starved vs. control ratio.

**Fig 8:**
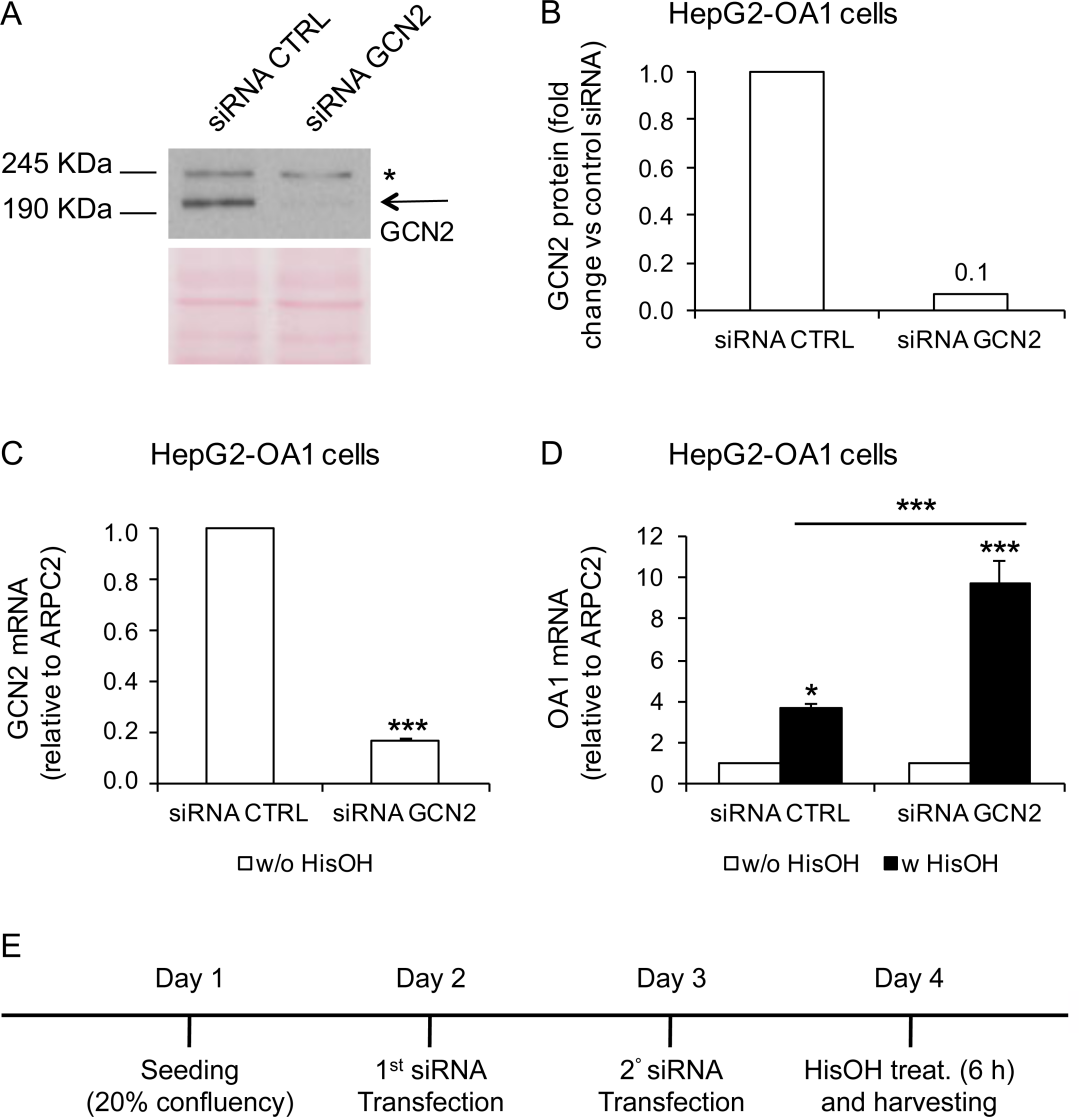
GCN2 knockdown does not interfere with transgene reactivation in HepG2 cells. (A, B) Downregulation of GCN2 protein by RNAi, shown by immunoblotting (A) and quantification (B) of protein extracts from HepG2-OA1 cells, transfected with control or anti-GCN2 siRNAs, and incubated with anti-GCN2 antibody. Arrow, GCN2 specific band; asterisk, non-specific signal detected by the Ab. Ponceau staining was used as loading control. Data are expressed as fold change vs. control siRNA (siRNA CTRL = 1). (C) Relative GCN2 mRNA abundance in HepG2-OA1 cells transfected with control or anti-GCN2 siRNAs. Mean ± SEM of 3 independent experiments. Data are expressed as fold change vs. control siRNA (siRNA CTRL = 1). ***P<0.001 (paired two-tailed Student’s t-test vs. control). (D) Relative transgene (OA1) mRNA abundance in HepG2-OA1 cells transfected with control or anti-GCN2 siRNAs and incubated for 6 h with L-Histidinol (HisOH, GCN2 activator; 4 mM). Mean ± SEM of 3 independent experiments. Data are expressed as fold change vs. untreated control (w/o HisOH = 1). *P<0.05, ***P<0.001 (one way ANOVA, followed by Tukey’s post-test; P values refer to comparisons vs. control, unless otherwise indicated). (E) Schematic representation of the experiment.

Similarly, we generated GCN2-KO clones from the parental HepG2-OA1 cell line by the same strategy. By using a guide RNA against exon 1 of the GCN2 gene, we obtained three independent GCN2-KO clones, namely E23, F22 and F27. Genomic characterization confirmed the presence of mutations on both alleles of exon 1 of the GCN2 gene in clone F27 (**S7 Fig**) and all three clones showed a very low amount—if any—of residual GCN2 protein, compared to the original HepG2-OA1 cell line (**Fig 7A**). To assess the ability of GCN2-KO cells to reactivate the transgene upon starvation, we cultured parental HepG2-OA1 cells and the three GCN2-KO clones in media deprived of Met/Cys or His (corresponding to the most effective treatments in this cell line; see **Fig 3B**) for 24 h, and evaluated the transgene expression by RT-qPCR. As shown in **Fig 7B and 7C**, we found that the reactivation of the OA1 transgene is neither abolished, nor reduced by KO of GCN2, as in HeLa cells. To further confirm this result, we knocked-down GCN2 by RNA interference (RNAi), and incubated the cells with or without L-Histidinol for 6 h. As shown in **Fig 8**, treatment of HepG2-OA1 cells with L-Histidinol results in efficient transgene reactivation, even upon significant GCN2 downregulation, both at the mRNA and protein levels. Taken together, these data strongly support the conclusion that GCN2 is not necessary for transgene reactivation in response to EAA starvation, either in HeLa or in HepG2 cells.

### MAPKs are required for the transgene reactivation response and ribosomal inhibition is sufficient to reproduce it

**Fig 9:**
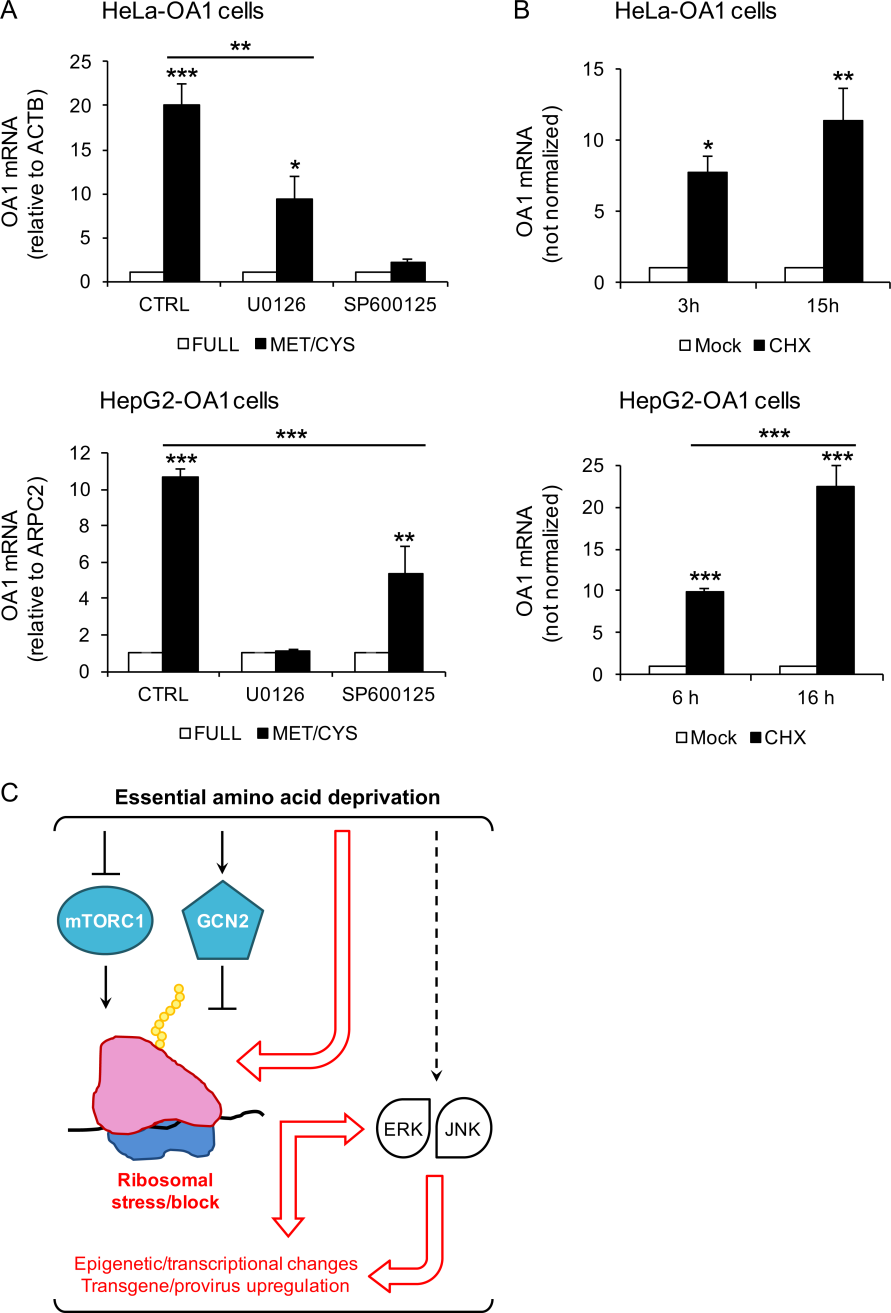
Transgene reactivation is abolished by MAPK inhibitors, and is induced by ribosomal inhibitors. (A) Relative transgene (OA1) mRNA abundance in HeLa-OA1 and HepG2-OA1 cells cultured in full medium or Met/Cys-deprived medium, in the presence or absence of inhibitors for MEK1/2 (U0126; 50 μM), and JNK1/2/3 (SP600125; 20–50 μM) for 24 h. For HeLa-OA1 cells data represent the mean ± SEM of 4 (CTRL and SP600125), or the mean ± range of 2 (U0126) independent experiments; for HepG2-OA1 cells, data represent the mean ± SEM of 3 independent experiments. Results are expressed as fold change vs. control (full medium = 1). *P<0.05, **P<0.01, ***P<0.001 (one way ANOVA, followed by Tukey’s post-test; P values refer to comparisons vs. control, unless otherwise indicated). Reference genes for qPCR: ACTB (actin beta; HeLa) and ARPC2 (HepG2). (B) Relative transgene (OA1) mRNA abundance in HeLa-OA1 and HepG2-OA1 cells cultured in the presence of CHX (protein elongation inhibitor; 50–100 ug/ml) for different time points, as indicated, compared to untreated control. For HeLa-OA1 cells, data represent the mean ± SEM of 3 independent experiments; for HepG2-OA1 cells, data represent the mean ± SEM of 4 (6 h), or the mean ± range of 2 (16 h) independent experiments. Results are expressed as fold change vs. control (mock = 1). *P<0.05, **P<0.01, ***P<0.001 (one way ANOVA, followed by Tukey’s post-test; P values refer to comparisons vs. control, unless otherwise indicated). Since the expression of reference genes used for normalizations change considerably upon CHX treatment (particularly at 15–16 h), the data presented are not normalized. However, comparable results were obtained by ARPC2 normalization. (C) Model for the transgene reactivation response to EAA starvation. EAA deficiency inhibits mTORC1 and activates GCN2, which attenuate general translation at different initiation steps. We propose the presence of an additional pathway (red arrows and text), thereby EAA limitation may directly lead to ribosomal stalling or delay during translation initiation (Met deficiency) and/or elongation (all EAA deficiencies), eventually resulting in epigenetic/transcriptional changes. The ERK and JNK branches of MAPKs are known to be activated during EAA starvation by yet unclear mechanisms.

Although GCN2 represents the most specific and characterized sensor of free tRNAs accumulation and AA limitation, gene expression during AA starvation is also regulated by additional poorly characterized mechanisms that are independent of GCN2 [17, 18, 55]. In particular, the Jun N-terminal Kinase (JNK) and MAPK/ERK Kinase (MEK)/Extracellular signal-Regulated Kinase (ERK) branches of the mitogen-activated protein kinases (MAPKs) are known to participate in the regulation of gene expression upon AA limitation in HeLa and HepG2 cells, respectively [48, 49, 56]. Thus, we tested whether their pharmacological inhibition may interfere with transgene reactivation by EAA starvation. As shown in **Fig 9A,** we observed that the JNK1/2/3 inhibitor SP600125 efficiently suppresses the reactivation of the OA1 transgene by Met/Cys-deprivation in HeLa-OA1 cells, while the MEK1/2 inhibitor U0126 has a less pronounced effect, consistent with the evidence for a prominent role of JNK in the AA response in HeLa cells [17]. By contrast, and again consistent with the literature [18], the inhibition of MEK1/2 completely suppresses the reactivation of the OA1 transgene by Met/Cys-deprivation in HepG2-OA1 cells, while inhibition JNK1/2/3 has only a partial effect. Comparable although milder effects were obtained with the MEK1 inhibitor PD98059 (not shown). These data suggest that the MEK/ERK and JNK pathways are necessary for transgene reactivation by EAA starvation.

In addition to activating GCN2, the presence of uncharged tRNAs—either due to AA deficiency or to the presence of aminoacyl-tRNA synthetase inhibitors (such as L-Histidinol)—is expected to result in protein synthesis impairment and ribosomal dysfunction. In fact, although less efficiently than charged tRNAs, uncharged tRNAs can enter the A-site of the ribosome, leading to ribosome stalling [57, 58]. To address the potential role of ribosomal impairment, we used Cycloheximide (CHX), a protein synthesis inhibitor that acts by directly blocking the translocation step during translation elongation [59, 60]. We found that the expression of the OA1 transgene increases promptly and substantially, after treatment of HeLa-OA1 and HepG2-OA1 cells with CHX for 3–6 or 15–16 h (**Fig 9B**). Treatment of HeLa-OA1 and HepG2-OA1 cells with another initiation/translocation inhibitor, Pactamycin [60], leads to comparable effects (not shown). Similar results were obtained with C2C12-GFP cells, despite a greater variability in the GFP transgene expression (CHX for 5–6 h led to increases of GFP mRNA of 450–2000 folds; 3 independent experiments). These findings indicate that pharmacological inhibitors of protein synthesis that directly target the ribosome can induce transgene reactivation similarly to, but more rapidly than EAA starvation.

## Discussion

In this work, we used the transcriptional reactivation of integrated and silenced transgenes in cultured cells to characterize a novel response to imbalanced EAA starvation, possibly leading to genome-wide epigenetic and/or transcriptional modifications. We show that this transcriptional response is reproducible in human and mouse cells, from either male (HepG2) or female (HeLa and C2C12), from different tissues (epithelium, liver, and skeletal muscle), either transformed (HeLa and HepG2) or immortalized (C2C12). Thus, it is conceivable that the transgene reactivation response to EAA starvation is part of a general adaptive change in transcriptional regulation, possibly affecting other somehow similar endogenous sequences, such as transposable elements. Consistently, we found that, among repeated elements, ERVs are preferentially upregulated upon Met/Cys starvation. This pattern of repeat expression is different from that observed in mammalian senescent cells and aging somatic tissues, where non-LTR retrotransposons (LINEs, SINEs and SVAs) were found to increase expression and ultimately transposition [61, 62].

ERVs, comprising 8% of the human genome, represent the remnants of past infections of germ cells by exogenous retroviruses, and are mostly unable to retrotranspose in the human genome [63]. However, they can reactivate during physiological development [64], or in pathological conditions like cancer, and regulate the expression of nearby genes by their LTR elements, leading to general transcriptional reprogramming [33, 63, 65]. In addition, epigenetic therapy in cancer has been found to upregulate the expression of ERVs, leading to accumulation of cytosolic double-stranded RNAs and consequent activation of the innate anti-viral defense pathway, which increases sensitivity to immunotherapy [66–69]. Thus, molecular characterization of the transgene reactivation response to EAA starvation may provide insights into the transcriptional regulation of these anciently-integrated mobile elements of the genome, with potential therapeutical applications.

Except for Trp, the transgene reactivation response appears specific for the limitation of any individual EAA, with quantitative differences among different EAAs and cell lines. Due to its ability to respond to deprivation of any EAA, GCN2 was initially considered as the most likely candidate to mediate the transgene reactivation. However, we found that neither GCN2, nor its downstream pathway are implicated in this process, since neither KO nor knock-down of GCN2 interfere with the response, and the ISR is neither necessary nor sufficient to trigger it. In addition, in agreement with previous results, neither the pharmacological inhibition of mTOR, nor the deprivation of serum elicited major effects (this work and [30]), supporting the notion that the response is not due to inhibition of the IGF1-mTORC1 axis. Overall, these negative results suggest that a novel EAA sensing and signaling pathway is involved.

The activity of the ribosome represents one of the most energy consuming processes in cells. Therefore, a common general response to nutrient restriction is translational repression, which consistently has been correlated to longevity [15, 70]. The inhibition of mTORC1 and the activation of GCN2/ISR are well known mechanisms responsible for general attenuation of protein synthesis in conditions of nutrient restriction, however, they are not implicated in the transgene reactivation response. By contrast, we found that protein synthesis inhibitors that act by directly blocking translation at the ribosome, such as CHX, are able to substantially and promptly upregulate the transgenes. Since the presence of free tRNAs can lead *per se* to translational block, or stalling [57, 58], we hypothesize that imbalanced EAA starvation directly leads to ribosomal stress/dysfunction, which in turn triggers the transgene reactivation response, possibly through a MAPK cascade (**Fig 9C**). Although preliminary genetic and pharmacological attempts did not allow us to define the precise function of ERK/JNK in the pathway, a central role for the ribosome would also explain why the deficiency of Trp, a rare AA in proteins, is able to activate GCN2/ISR, yet consistently results in no or minimal reactivation of the transgenes, since its absence would presumably affect translation less than other EAAs.

A well characterized signaling pathway triggered by ribosomal stalling is the stringent response in bacteria, which links nutrient starvation with all levels of gene expression. Upon stressful stimuli, especially the shortage of AAs, the survival and virulence of bacteria are preserved by a general transcriptional/translational response, triggered by RelA/SpoT. This enzyme specifically recognizes ribosomes stalled with uncharged tRNA [71], and synthesize pleiotropic second messengers guanosine tetraphosphate and guanosine pentaphosphate (collectively referred to as (p)ppGpp), leading to a variety of transcriptional and translational changes [72, 73]. In metazoa, AA unavailability also induces ribosome stalling, and in turn activates the ribosome-associated protein quality control pathway, which interrupts protein synthesis and targets mRNAs and proteins for degradation, when the translation process is (assumed to be) corrupted [58]. It is possible that, by inducing ribosomal block or delay, during translation initiation and/or elongation, EAA limitation in mammalian cells triggers additional signaling pathways than presently known. Alternatively, translation inhibition may determine downregulation of specific epigenetic regulators that are unstable and therefore require a high translation rate. Interestingly, the general translational output has been recently reported to modulate chromatin accessibility in embryonic stem cells, since either mTOR, or ribosomal inhibitors are able to decrease it at active developmental enhancers, while they increase it at histone genes and transposable elements [74].

Overall, our findings indicate the presence of a novel transcriptional response to EAA limitation, which affects the expression of exogenous non-native DNA sequences, and may similarly involve endogenous genomic retroelements. Dissection of the underlying mechanism ruled out a role for the main AA-deficiency sensor GCN2 and pointed to the ribosome as the possible master controller. Given the roles of nutrient and AA restriction in human health and longevity, these findings may contribute to our understanding of the epigenetic/transcriptional mechanisms operating in aging and age-related diseases, and suggest nutritional or pharmacological strategies to prevent or control them. In addition, our model mimic in an extreme way the partial AA deficiencies that may derive from imbalanced nutrition, since the full balanced AA complement is lacking in some common plant sources of dietary proteins, including wheat, legumes, grains and rice (naturally deficient in Met and/or Lys) [15, 18]. It will be of interest to determine whether also partial EAA deficiencies can result in similar epigenetic/transcriptional modifications, as well as whether in conditions of partial Met deficiency (rather than complete absence, like in our setting) the presence of Cys is able to compensate, as demonstrated in rodents *in vivo* for the physiological effects of dietary Met restriction [75].

## Supporting information

S1 Fig Upregulation of endogenous retroviruses upon Met/Cys starvation in HeLa cells. (A) Classification of genomic repeats belonging to differential expression clusters 1 to 4 upon Met/Cys starvation. Clusters 1 and 2 include upregulated repeats, while Clusters 3 and 4 include downregulated sequences. (B) Class distribution of repeat subfamilies, either significantly upregulated or downregulated upon starvation, compared to all genomic repeat subfamilies (first column). Class DNA includes DNA transposons; SINE includes Alu; LINE includes L1 an L2; LTR includes endogenous retroviruses and solitary LTRs; Satellite includes centromeric acrosomal and telomeric satellites; Others includes SVA, simple repeats, snRNA, and tRNAs. LTR-retroelements are significantly enriched among repeats that are upregulated upon starvation, while LINEs are significantly enriched among repeats that are downregulated. ***P<0.001 (Fisher exact test).
(PDF)

S2 Fig Enrichment analysis of genomic repeats upon Met/Cys starvation in HeLa cells. (A) Enrichment analysis, performed per class or family, of genomic repeats belonging to differential expression clusters 1–4 (see Methods for details). (B) Enrichment analysis, performed per class, of genomic repeats significantly up- or downregulated upon starvation (see Methods for details). Both Odds-Ratios and P values are indicated (Fisher Exact test).
(PDF)

S3 Fig Transgene reactivation by EAA deprivation and by the pan HDAC inhibitor TSA in HeLa and HepG2 cells. (A) Relative transgene (GFP) and CHOP mRNA abundance in HeLa-GFP cells, cultured in Met/Cys-deprived medium or in the presence of TSA for 24 h, compared to full medium. Mean ± SD of 3 technical replicates from 1 experiment. (B) Relative transgene (OA1) and CHOP mRNA abundance in HepG2-OA1 cells, cultured in Met/Cys-deprived medium or in the presence of TSA for 16 h, compared to full medium. Mean ± SD of 3 technical replicates from 1 experiment representative of two. (C) Relative transgene (GFP) mRNA abundance in HeLa-GFP cells, cultured in various AA-deprived media for 48 h, compared to full medium. Mean ± SD of 3 technical replicates from 1 experiment. The high degree of reactivation in HeLa-GFP cells probably depends on the high number of plasmids integrated into the genome of this specific clone. (D) Immunoblotting of protein extracts from HeLa-OA1 and HepG2-OA1 cells, starved for 48 and 24 h, respectively, in Met/Cys, Cys or Met deficient media, compared to full medium. Arrows indicate the specific bands corresponding to the OA1 transgenic protein. Ponceau staining was used as loading control. (E) Relative transgene (OA1) mRNA abundance in HeLa-OA1 and HepG2-OA1 cells, cultured in Met/Cys, Cys only, or Met only deficient media for 48 h and 24 h, respectively, compared to full medium. Mean ± SEM of 3 independent experiments. All qPCR data are expressed as fold change vs. control (full medium = 1). ***P<0.001 (one way ANOVA, followed by Tukey’s post-test; P values refer to comparisons vs. control, unless otherwise indicated).
(PDF)

S4 Fig Transgene reactivation by EAA deprivation and by the pan HDAC inhibitor TSA in C2C12 mouse cells. (A) Relative transgene (GFP) mRNA abundance in C2C12-GFP cells, following treatment with TSA for 24 h, compared to untreated cells. Mean ± SEM of 4 independent experiments. Data are expressed as fold change vs. control (full medium = 1). **P = 0.0011 (paired two-tailed Student’s t-test vs. control). (B) Relative transgene (GFP) and CHOP mRNA abundance in C2C12-GFP cells cultured in various AA-deprived media for 24 h, compared to full medium. Mean ± SEM of 3 independent experiments. Data are expressed as fold change vs. control (full medium = 1). *P<0.05, **P<0.01, ***P<0.001 (one way ANOVA, followed by Dunnett’s post-test vs. full medium). The high degree of reactivation in C2C12-GFP cells probably depends on the high number of plasmids integrated into the genome of this specific clone, and determines a wide variability of transgene expression in different conditions.
(PDF)

S5 Fig Transgene expression upon mTOR inhibition or GCN2 activation in HeLa and C2C12 cells. Relative transgene (GFP) and CHOP mRNA abundance in HeLa-GFP (A) and C2C12-GFP (B) cells, cultured in Met/Cys-deprived medium, or in the presence of PP242 (mTOR inhibitor; 1–3 μM) or L-Histidinol (HisOH, GCN2 activator; 4–16 mM), either alone or in combination for 24 h, compared to full medium. Mean ± SD of 3 technical replicates from 1 experiment (A), or mean ± SEM of 3 independent experiments (B). Data are expressed as fold change vs. control (full medium = 1). **P<0.01, ***P<0.001 (one way ANOVA, followed by Dunnett’s post-test vs. full medium).
(PDF)

S6 Fig Characterization of GCN2 genomic editing by CRISPR/Cas9 technology in HeLa cells. (A) PCR amplification of GCN2 exon 1 and 6 from HeLa-OA1 parental cells and GCN2-KO clones 183#11 and 185#5 (targeted at exon 1), and 239 #1 (targeted at exon 6). The 185#5 clone contains multiple alleles, suggestive of a mixed cell population. (B) Sequence of GCN2 exon 1 in HeLa-OA1 parental cells (wild type) and GCN2-KO clone 183#11, showing a single base deletion. (C) Semi-quantitative PCR obtained by simultaneously amplifying GCN2 exon 1 (and exon 6 as normalization control) from HeLa-OA1 parental cells and GCN2-KO clone 183#11. The clone shows a significant amplification delay of exon 1, consistent with a deletion. Relative band density is shown below. (D) Sequence of GCN2 exon 6 in HeLa-OA1 parental cells (wild type) and GCN2-KO clone 239#1, showing a single base insertion and a 48 bp deletion in the clone. The original guide sequence is shown in bold red.
(PDF)

S7 Fig Characterization of GCN2 genomic editing by CRISPR/Cas9 technology in HepG2 cells. (A) PCR amplification of GCN2 exon 1 from HepG2-OA1 parental cells and GCN2-KO clone F27 (targeted at exon 1). (B) Sequence of GCN2 exon 1 in HepG2-OA1 parental cells (wild type) and GCN2-KO clone F27, showing a two bp insertion for allele 1 and an 18 bp deletion for allele 2. The original guide sequence is shown in bold red.
(PDF)

S1 Table Formula of full, DMEM-based, medium. (XLSX)

S2 Table gRNAs for CRISPR/Cas9 & Primers for PCR. (XLSX)

S1 File Gene set enrichment analysis using Gene Ontology Biological Process, KEGG and Reactome databases. (XLSX)
