# Supplementary material for: Amino acid deprivation triggers a novel GCN2-independent response leading to the transcriptional reactivation of non-native DNA sequences
Source: PLoS One. 2018 Jul 18;13(7):e0200783. doi: 10.1371/journal.pone.0200783 (PMC6051655; doi:10.1371/journal.pone.0200783)

S1 Fig

A

Cluster 1
UP-regulated progressively

Repeat Name	Repeat Family	Repeat Class
Charlie10b	hAT-Charlie	DNA
Charlie1	hAT-Charlie	DNA
MER45B	hAT-Tip100	DNA
L1M2b	L1	LINE
L1M3b	L1	LINE
L1M3f	L1	LINE
L1P4d	L1	LINE
Harlequin-int	ERV1	LTR
HERV15-int	ERV1	LTR
HERVK14-int	ERVK	LTR
HERVL18-int	ERVL	LTR
HERVL-int	ERVL	LTR
LOR1-int	ERV1	LTR
LTR12B	ERV1	LTR
LTR17	ERV1	LTR
LTR39	ERV1	LTR
LTR41B	ERVL	LTR
LTR51	ERV1	LTR
LTR61	ERV1	LTR
LTR7	ERV1	LTR
LTR77	ERV1	LTR
LTR78	ERV1	LTR
LTR82A	ERVL	LTR
LTR8	ERV1	LTR
LTR85b	Gypsy?	LTR
LTR86B2	ERVL	LTR
LTR88a	Gypsy?	LTR
LTR9	ERV1	LTR
MamGyp-int	Gypsy	LTR
MER110A	ERV1	LTR
MER110-int	ERV1	LTR
MER41E	ERV1	LTR
MER4-int	ERV1	LTR
MER50B	ERV1	LTR
MER52D	ERV1	LTR
MER57-int	ERV1	LTR
MER65-int	ERV1	LTR
MER67C	ERV1	LTR
MER84-int	ERV1	LTR
MER87	ERV1	LTR
MLT1A	ERVL-MaLR	LTR
MLT1A-int	ERVL-MaLR	LTR
MLT1E1	ERVL-MaLR	LTR
MLT1F1-int	ERVL-MaLR	LTR
MLT1F	ERVL-MaLR	LTR
MLT1H1-int	ERVL-MaLR	LTR
MLT1H-int	ERVL-MaLR	LTR
MLT1J1-int	ERVL-MaLR	LTR
MSTB2-int	ERVL-MaLR	LTR
MSTB-int	ERVL-MaLR	LTR
PrimLTR79	ERV1	LTR
THE1A	ERVL-MaLR	LTR
ACRO1	acro	Satellite
ALR/Alpha	centr	Satellite
CER	Satellite	Satellite
SATR2	Satellite	Satellite
(CAGT)n	Simple_repeat	Simple_repeat
(CTCA)n	Simple_repeat	Simple_repeat

Cluster 2
UP-regulated at early time points

Repeat Name	Repeat Family	Repeat Class
Arthur1A	hAT-Tip100	DNA
Charlie3	hAT-Charlie	DNA
Charlie6	hAT-Charlie	DNA
Tigger5	TcMar-Tigger	DNA
L1MA5	L1	LINE
HERVK13-int	ERVK	LTR
HUERS-P3-int	ERV1	LTR
LTR14B	ERVK	LTR
LTR16A1	ERVL	LTR
LTR27B	ERV1	LTR
LTR30	ERV1	LTR
LTR33	ERVL	LTR
LTR4	ERV1	LTR
LTR5_Hs	ERVK	LTR
LTR76	ERV1	LTR
LTR89	ERVL?	LTR
MER34A1	ERV1	LTR
MER68B	ERVL	LTR
MER87B	ERV1	LTR
MLT1E1A	ERVL-MaLR	LTR
PRIMA41-int	ERV1	LTR
HSAT4	centr	Satellite
(CTGGGG)n	Simple_repeat	Simple_repeat
tRNA-Gln-CAA	tRNA	tRNA
tRNA-Gly-GGA	tRNA	tRNA
tRNA-Ser-TCA	tRNA	tRNA
UCON4	Unknown	Unknown

Cluster 3
DOWN-regulated progressively

Repeat Name	Repeat Family	Repeat Class
MER106A	hAT-Charlie	DNA
MER30	hAT-Charlie	DNA
MER94	hAT-Blackjack	DNA
Tigger10	TcMar-Tigger	DNA
Tigger16b	TcMar-Tigger	DNA
Tigger4b	TcMar-Tigger	DNA
Tigger9a	TcMar-Tigger	DNA
UCON23	DNA?	DNA?
AluSc	Alu	SINE
CR1_Mam	CR1	LINE
L1M3	L1	LINE
L2b	L2	LINE
L3b	CR1	LINE
L4	RTE	LINE
LTR19A	ERV1	LTR
LTR32	ERVL	LTR
LTR35	ERV1	LTR
LTR72B	ERV1	LTR
LTR90A	LTR	LTR
MSTD	ERVL-MaLR	LTR
LSU-rRNA_Hsa	rRNA	rRNA
(CAAAA)n	Simple_repeat	Simple_repeat
(CAGGG)n	Simple_repeat	Simple_repeat
(CAT)n	Simple_repeat	Simple_repeat
(CCTTG)n	Simple_repeat	Simple_repeat
(CTTG)n	Simple_repeat	Simple_repeat
(TA)n	Simple_repeat	Simple_repeat
(TATAG)n	Simple_repeat	Simple_repeat
U17	snRNA	snRNA
UCON22	Unknown	Unknown

Cluster 4
DOWN-regulated at late time points

Repeat Name	Repeat Family	Repeat Class
Charlie24	hAT-Charlie	DNA
Cheshire	hAT-Charlie	DNA
DNA1_Mam	TcMar	DNA
MER75A	PiggyBac	DNA
MER91B	hAT-Tip100	DNA
MER97b	hAT-Tip100	DNA
Tigger13a	TcMar-Tigger	DNA
Tigger2b_Pri	TcMar-Tigger	DNA
AluYh9	Alu	SINE
HAL1	L1	LINE
L1M2	L1	LINE
L1M3de	L1	LINE
L1M4	L1	LINE
L1M5	L1	LINE
L1MA2	L1	LINE
L1MA3	L1	LINE
L1MA6	L1	LINE
L1MA9	L1	LINE
L1MB1	L1	LINE
L1MB2	L1	LINE
L1MB3	L1	LINE
L1MC1	L1	LINE
L1MD2	L1	LINE
L1ME1	L1	LINE
L1ME2z	L1	LINE
L1ME3F	L1	LINE
L1ME5	L1	LINE
L1MEa	L1	LINE
L1MEc	L1	LINE
L1MEe	L1	LINE
L1MEg	L1	LINE
L1MEg2	L1	LINE
L1P5	L1	LINE
L1PA14	L1	LINE
L1PB3	L1	LINE
L1PB4	L1	LINE
Mam_R4	Dong-R4	LINE
HERVI-int	ERV1	LTR
LTR16B	ERVL	LTR
LTR57	ERVL	LTR
LTR82B	ERVL	LTR
MER4B-int	ERV1	LTR
MER52A	ERV1	LTR
MER89-int	ERV1	LTR
MLT1G3-int	ERVL-MaLR	LTR
(ATAGG)n	Simple_repeat	Simple_repeat
(ATGTG)n	Simple_repeat	Simple_repeat
(CACAT)n	Simple_repeat	Simple_repeat
(CACCT)n	Simple_repeat	Simple_repeat
(CACTA)n	Simple_repeat	Simple_repeat
(CATA)n	Simple_repeat	Simple_repeat
(CATAT)n	Simple_repeat	Simple_repeat
(CATTG)n	Simple_repeat	Simple_repeat
(TCTA)n	Simple_repeat	Simple_repeat
tRNA-Ile-ATA	tRNA	tRNA
UCON5	Unknown	Unknown
UCON6	Unknown	Unknown

B

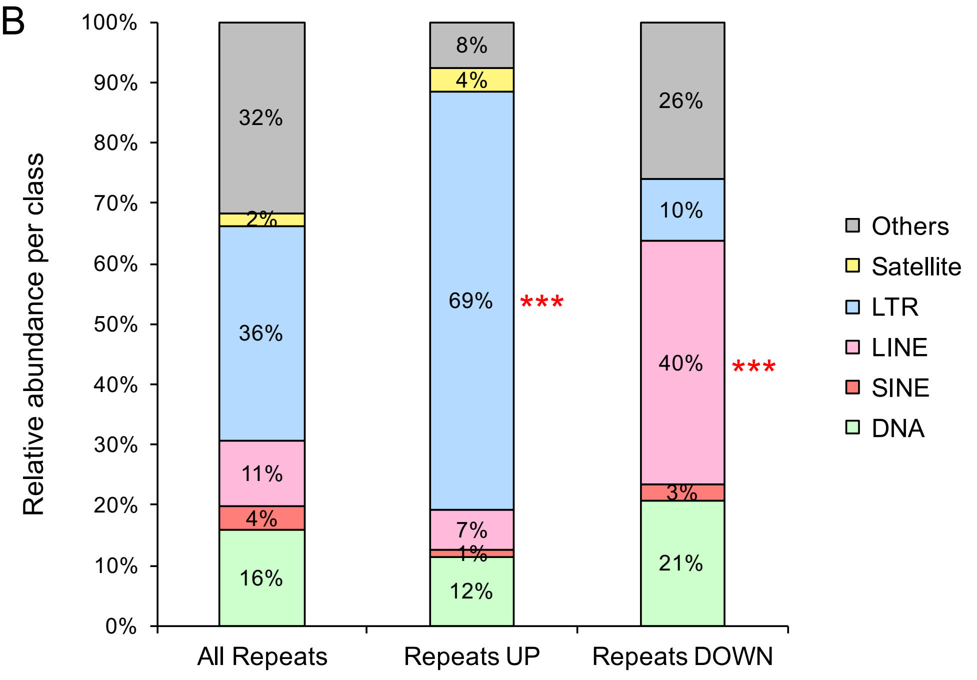

Supplement: S1 Fig — (A) Classification of genomic repeats belonging to differential expression clusters 1 to 4 upon Met/Cys starvation. Clusters 1 and 2 include upregulated repeats, while Clusters 3 and 4 include downregulated sequences. (B) Class distribution of repeat subfamilies, either significantly upregulated or downregulated upon starvation, compared to all genomic repeat subfamilies (first column). Class DNA includes DNA transposons; SINE includes Alu; LINE includes L1 an L2; LTR includes endogenous retroviruses and solitary LTRs; Satellite includes centromeric acrosomal and telomeric satellites; Others includes SVA, simple repeats, snRNA, and tRNAs. LTR-retroelements are significantly enriched among repeats that are upregulated upon starvation, while LINEs are significantly enriched among repeats that are downregulated. ***P<0.001 (Fisher exact test). (PDF) [file pone.0200783.s001.pdf]
