# Supplementary material for: Amino acid deprivation triggers a novel GCN2-independent response leading to the transcriptional reactivation of non-native DNA sequences
Source: PLoS One. 2018 Jul 18;13(7):e0200783. doi: 10.1371/journal.pone.0200783 (PMC6051655; doi:10.1371/journal.pone.0200783)
Supplement: S2 Fig — (A) Enrichment analysis, performed per class or family, of genomic repeats belonging to differential expression clusters 1–4 (see Methods for details). (B) Enrichment analysis, performed per class, of genomic repeats significantly up- or downregulated upon starvation (see Methods for details). Both Odds-Ratios and P values are indicated (Fisher Exact test). (PDF) [file pone.0200783.s002.pdf]

S2 Fig

A

Repeat Class Enrichment in Clusters	Cluster 1 OR	Cluster 1 p-value	Cluster 2 OR	Cluster 2 p-value	Cluster 3 OR	Cluster 3 p-value	Cluster 4 OR	Cluster 4 p-value
DNA	0,32	5,31E-02	0,91	1,00E+00	1,65	2,34E-01	0,84	8,56E-01
DNA?	0,00	1,00E+00	0,00	1,00E+00	2,11	3,94E-01	0,00	1,00E+00
**LINE**	0,67	6,53E-01	0,35	5,09E-01	1,58	3,77E-01	4,83	1,91E-09
**LTR**	2,13	3,50E-04	1,64	1,20E-01	0,55	2,51E-01	0,37	6,63E-03
rRNA	0,00	1,00E+00	0,00	1,00E+00	15,50	8,41E-02	0,00	1,00E+00
**Satellite**	4,26	2,26E-02	2,25	3,77E-01	0,00	1,00E+00	0,00	1,00E+00
Simple_repeat	0,16	1,46E-03	0,17	4,58E-02	1,05	8,32E-01	0,69	3,38E-01
SINE	0,00	2,58E-01	0,00	1,00E+00	0,93	1,00E+00	0,47	7,19E-01
snRNA	0,00	1,00E+00	0,00	1,00E+00	3,88	2,48E-01	0,00	1,00E+00
tRNA	0,00	1,69E-01	2,46	1,45E-01	0,00	6,35E-01	0,38	5,13E-01
Unknown	0,00	6,27E-01	1,72	4,56E-01	1,55	4,90E-01	1,58	3,81E-01

Repeat Family Enrichment in Clusters	Cluster 1 OR	Cluster 1 p-value	Cluster 2 OR	Cluster 2 p-value	Cluster 3 OR	Cluster 3 p-value	Cluster 4 OR	Cluster 4 p-value
acro	24,47	7,82E-02	0,00	1,00E+00	0,00	1,00E+00	0,00	1,00E+00
Alu	0,00	4,02E-01	0,00	1,00E+00	1,13	5,97E-01	0,58	1,00E+00
centr	4,08	2,48E-01	8,61	1,30E-01	0,00	1,00E+00	0,00	1,00E+00
CR1	0,00	1,00E+00	0,00	1,00E+00	6,20	5,27E-02	0,00	1,00E+00
DNA	0,32	5,31E-02	0,91	1,00E+00	1,65	2,34E-01	0,84	8,56E-01
DNA?	0,00	1,00E+00	0,00	1,00E+00	2,11	3,94E-01	0,00	1,00E+00
Dong-R4	0,00	1,00E+00	0,00	1,00E+00	0,00	1,00E+00	23,64	8,07E-02
**ERV1**	2,35	1,11E-03	1,59	2,44E-01	0,54	4,65E-01	0,36	4,84E-02
**ERVK**	0,66	1,00E+00	4,19	4,62E-02	0,00	1,00E+00	0,00	3,98E-01
**ERVL**	2,02	2,46E-02	1,42	4,10E-01	0,51	5,72E-01	0,52	3,07E-01
ERVL?	0,00	1,00E+00	25,83	5,78E-02	0,00	1,00E+00	0,00	1,00E+00
**ERVL-MaLR**	3,06	4,33E-03	0,65	1,00E+00	0,58	1,00E+00	0,30	3,68E-01
Gypsy	3,86	5,69E-02	0,00	1,00E+00	0,00	1,00E+00	0,00	1,00E+00
**Gypsy?**	8,16	3,84E-02	0,00	1,00E+00	0,00	1,00E+00	0,00	1,00E+00
hAT	0,67	7,96E-01	1,42	4,77E-01	1,28	7,29E-01	0,87	1,00E+00
hAT-Blackjack	0,00	1,00E+00	0,00	1,00E+00	5,81	1,79E-01	0,00	1,00E+00
hAT-Charlie	0,72	1,00E+00	1,52	6,43E-01	1,37	6,59E-01	0,70	1,00E+00
hAT-Tip100	1,02	1,00E+00	2,15	3,89E-01	0,00	1,00E+00	1,97	2,91E-01
**L1**	0,80	1,00E+00	0,42	7,21E-01	0,38	5,08E-01	5,62	1,77E-10
L2	0,00	1,00E+00	0,00	1,00E+00	11,63	1,04E-01	0,00	1,00E+00
**LTR**	2,13	3,50E-04	1,64	1,20E-01	0,55	2,51E-01	0,37	6,63E-03
PiggyBac	0,00	1,00E+00	0,00	1,00E+00	0,00	1,00E+00	3,94	2,55E-01
rRNA	0,00	1,00E+00	0,00	1,00E+00	15,50	8,41E-02	0,00	1,00E+00
RTE	0,00	1,00E+00	0,00	1,00E+00	11,63	1,04E-01	0,00	1,00E+00
**Satellite**	4,26	2,26E-02	2,25	3,77E-01	0,00	1,00E+00	0,00	1,00E+00
Simple_repeat	0,16	1,46E-03	0,17	4,58E-02	1,05	8,32E-01	0,69	3,38E-01
SINE	0,00	2,58E-01	0,00	1,00E+00	0,93	1,00E+00	0,47	7,19E-01
snRNA	0,00	1,00E+00	0,00	1,00E+00	3,88	2,48E-01	0,00	1,00E+00
TcMar	0,00	1,08E-01	0,73	1,00E+00	2,62	8,59E-02	1,00	1,00E+00
**TcMar-Tigger**	0,00	2,60E-01	0,99	1,00E+00	3,58	3,66E-02	0,91	1,00E+00
tRNA	0,00	1,69E-01	2,46	1,45E-01	0,00	6,35E-01	0,38	5,13E-01
Unknown	0,00	6,27E-01	1,72	4,56E-01	1,55	4,90E-01	1,58	3,81E-01

OR = Odds-Ratio (>1, enriched; <1, depleted); p-value = Fisher exact test

Green = Significantly Enriched

Red = Significantly Depleted

Repeat name in bold = significantly enriched in at least one cluster

Only repeat families that are differentially expressed in one or more clusters are shown

B

Repeat Class Enrichment	UP-regulated repeats OR	UP-regulated repeats p-value	DOWN-regulated repeats OR	DOWN-regulated repeats p-value
DNA	0,7125	0,3233	1,4601	0,1959
SINE	0,2665	0,2530	0,7472	1,0000
**LINE**	0,6433	0,3101	7,2504	0,0000
**LTR**	4,6247	0,0000	0,2076	0,0000
Satellite	2,8884	0,0702	0,0000	0,6287
Others	0,1818	0,0000	0,7870	0,4458

OR = Odds-Ratio (>1, enriched; <1, depleted); p-value = Fisher exact test

Green = Significantly Enriched

Red = Significantly Depleted

Repeat name in bold = significantly enriched in at least one group (UP/DOWN)

Only main repeat classes are shown; Others includes SVA, simple repeats, snRNA, and tRNAs
