# Supplementary figures and images for: Amino acid deprivation triggers a novel GCN2-independent response leading to the transcriptional reactivation of non-native DNA sequences
Source: PLoS One. 2018 Jul 18;13(7):e0200783. doi: 10.1371/journal.pone.0200783 (PMC6051655; doi:10.1371/journal.pone.0200783)

S3 Fig

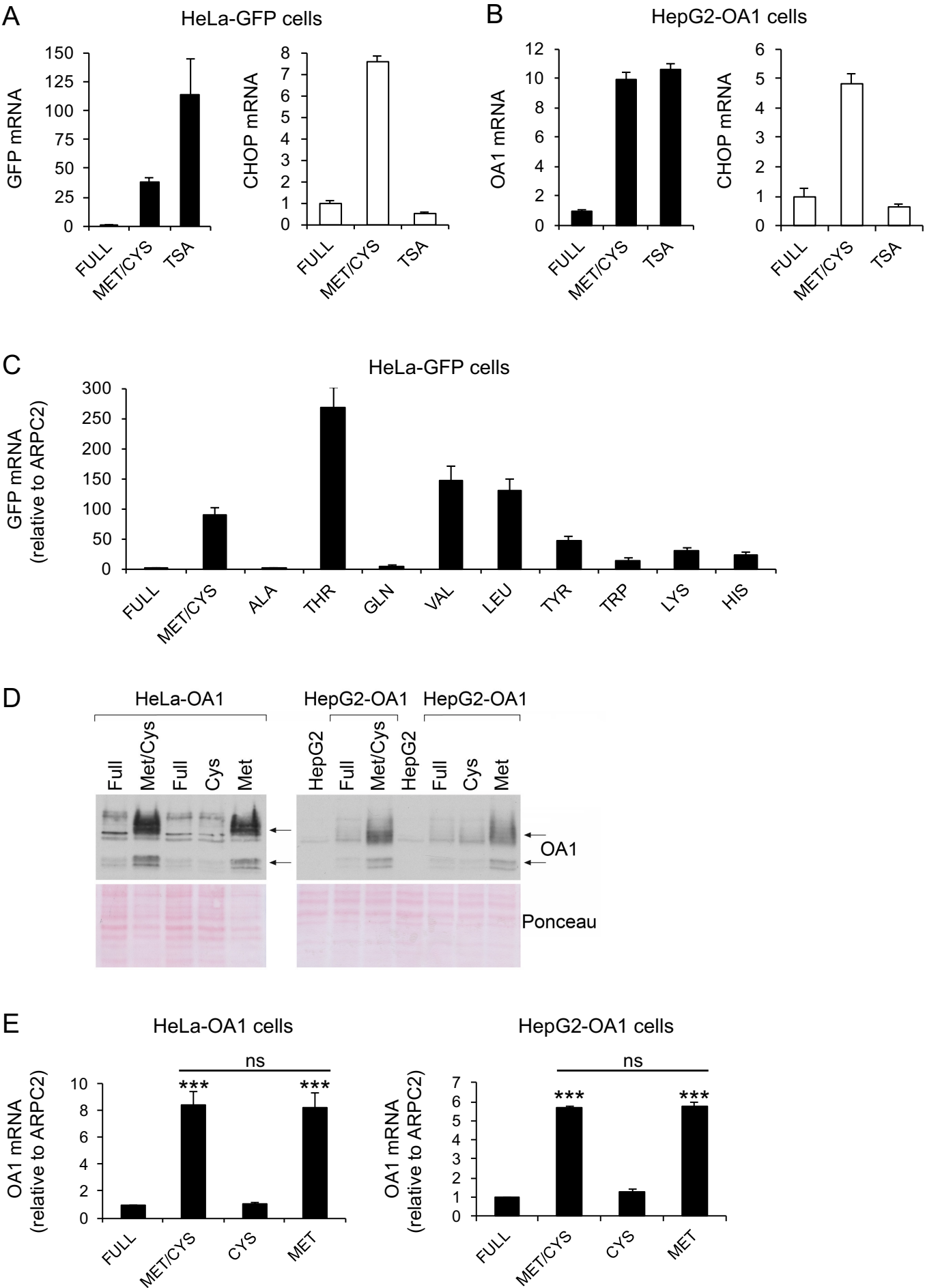

Supplement: S3 Fig — (A) Relative transgene (GFP) and CHOP mRNA abundance in HeLa-GFP cells, cultured in Met/Cys-deprived medium or in the presence of TSA for 24 h, compared to full medium. Mean ± SD of 3 technical replicates from 1 experiment. (B) Relative transgene (OA1) and CHOP mRNA abundance in HepG2-OA1 cells, cultured in Met/Cys-deprived medium or in the presence of TSA for 16 h, compared to full medium. Mean ± SD of 3 technical replicates from 1 experiment representative of two. (C) Relative transgene (GFP) mRNA abundance in HeLa-GFP cells, cultured in various AA-deprived media for 48 h, compared to full medium. Mean ± SD of 3 technical replicates from 1 experiment. The high degree of reactivation in HeLa-GFP cells probably depends on the high number of plasmids integrated into the genome of this specific clone. (D) Immunoblotting of protein extracts from HeLa-OA1 and HepG2-OA1 cells, starved for 48 and 24 h, respectively, in Met/Cys, Cys or Met deficient media, compared to full medium. Arrows indicate the specific bands corresponding to the OA1 transgenic protein. Ponceau staining was used as loading control. (E) Relative transgene (OA1) mRNA abundance in HeLa-OA1 and HepG2-OA1 cells, cultured in Met/Cys, Cys only, or Met only deficient media for 48 h and 24 h, respectively, compared to full medium. Mean ± SEM of 3 independent experiments. All qPCR data are expressed as fold change vs. control (full medium = 1). ***P<0.001 (one way ANOVA, followed by Tukey’s post-test; P values refer to comparisons vs. control, unless otherwise indicated). (PDF) [file pone.0200783.s003.pdf]

S4 Fig

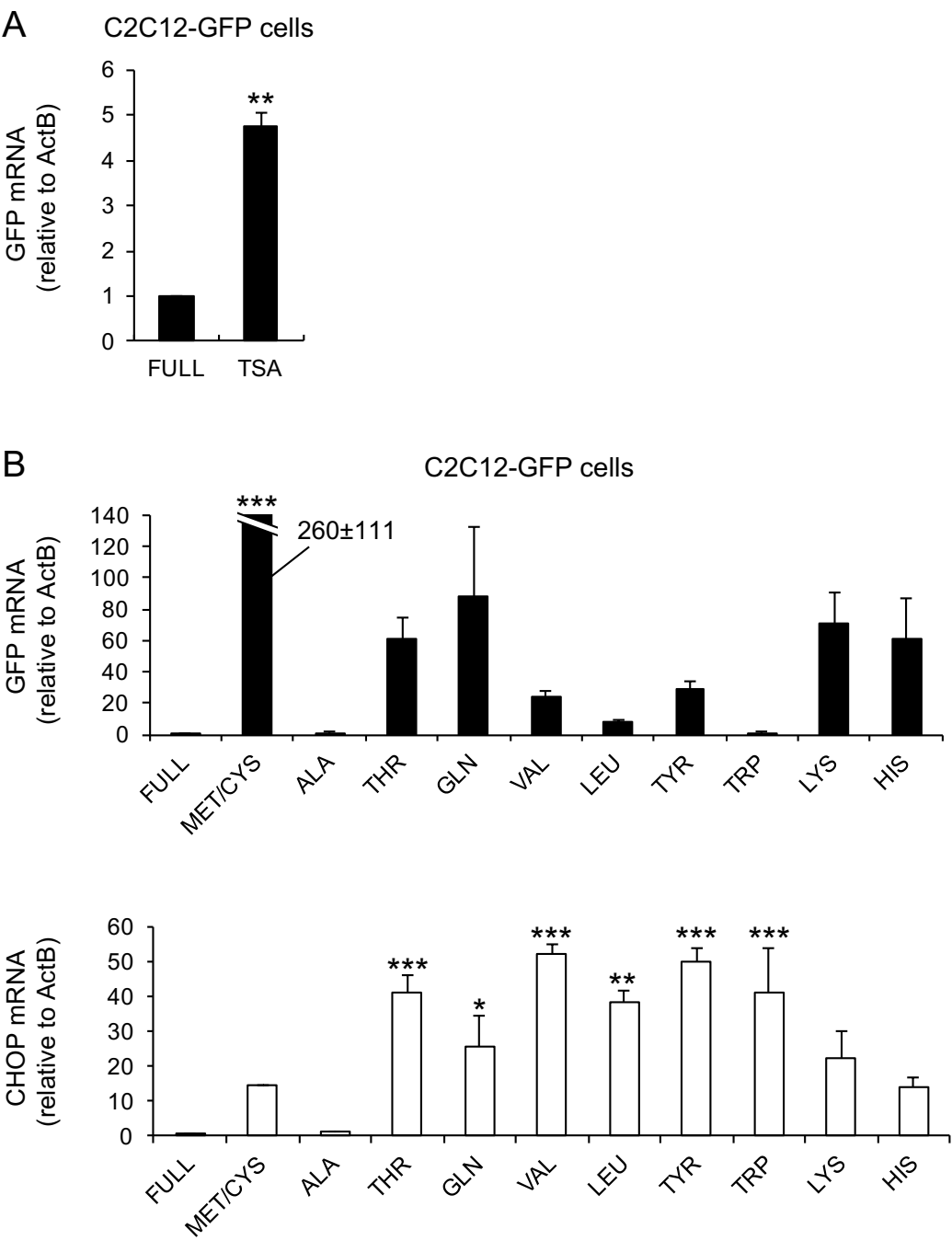

Supplement: S4 Fig — (A) Relative transgene (GFP) mRNA abundance in C2C12-GFP cells, following treatment with TSA for 24 h, compared to untreated cells. Mean ± SEM of 4 independent experiments. Data are expressed as fold change vs. control (full medium = 1). **P = 0.0011 (paired two-tailed Student’s t-test vs. control). (B) Relative transgene (GFP) and CHOP mRNA abundance in C2C12-GFP cells cultured in various AA-deprived media for 24 h, compared to full medium. Mean ± SEM of 3 independent experiments. Data are expressed as fold change vs. control (full medium = 1). *P<0.05, **P<0.01, ***P<0.001 (one way ANOVA, followed by Dunnett’s post-test vs. full medium). The high degree of reactivation in C2C12-GFP cells probably depends on the high number of plasmids integrated into the genome of this specific clone, and determines a wide variability of transgene expression in different conditions. (PDF) [file pone.0200783.s004.pdf]

S5 Fig

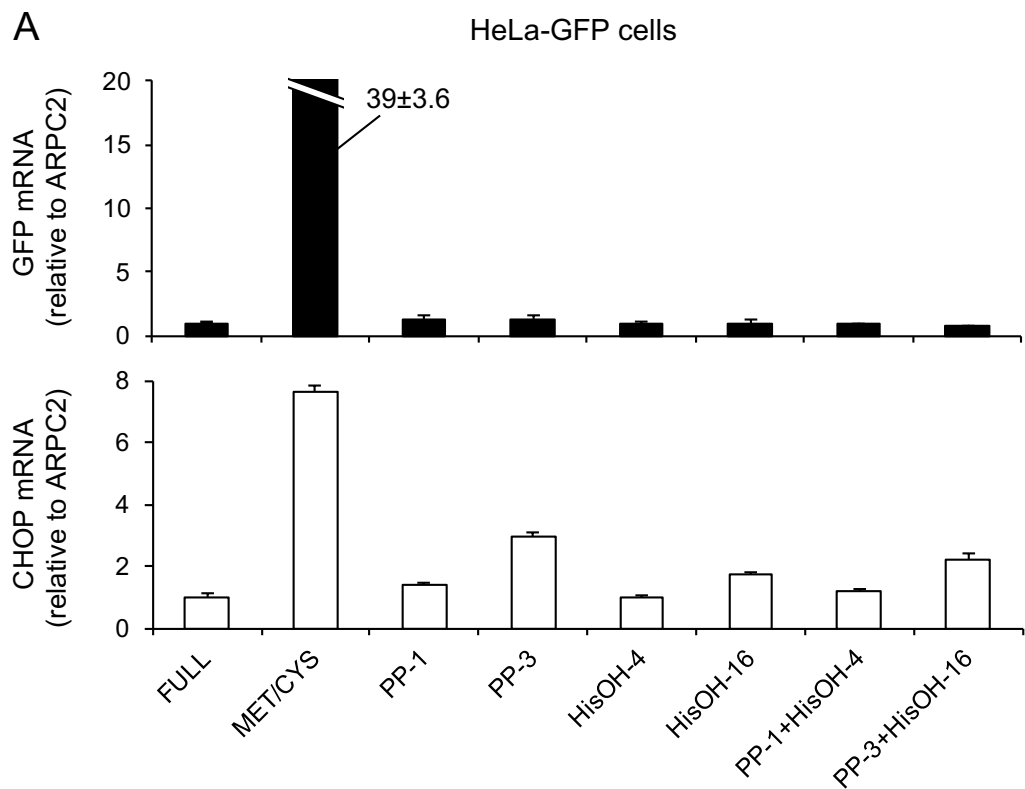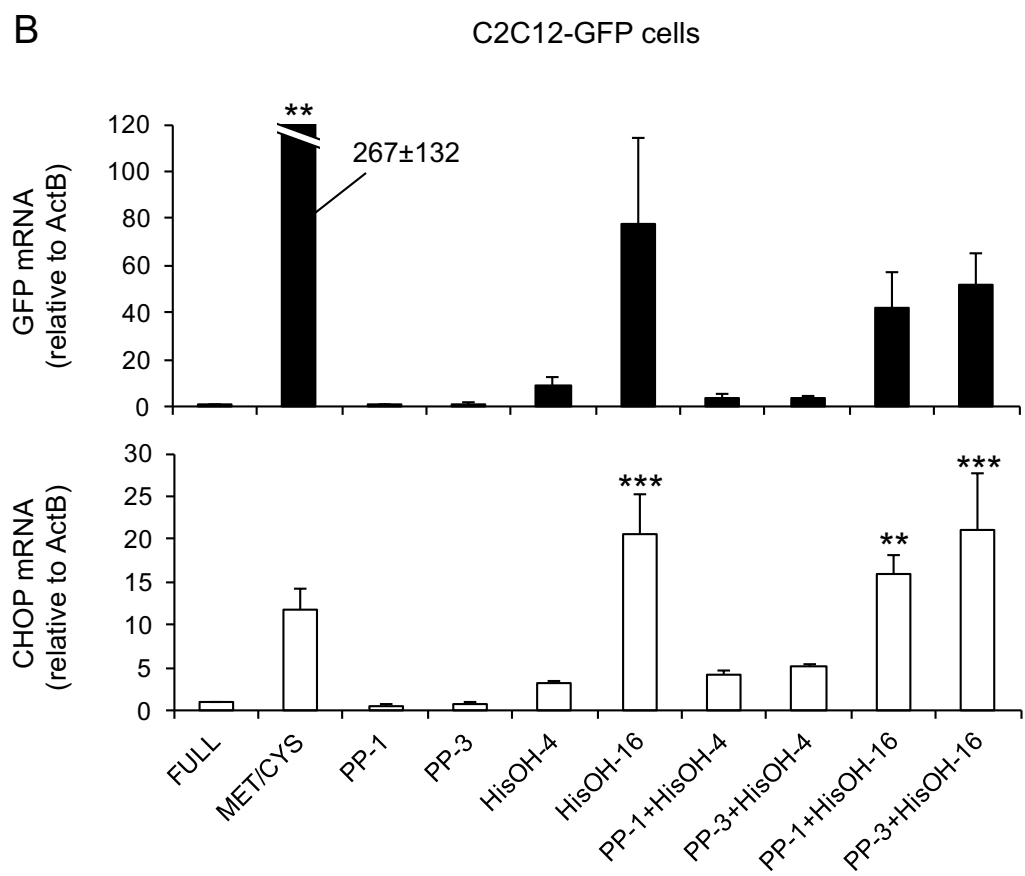

Supplement: S5 Fig — Relative transgene (GFP) and CHOP mRNA abundance in HeLa-GFP (A) and C2C12-GFP (B) cells, cultured in Met/Cys-deprived medium, or in the presence of PP242 (mTOR inhibitor; 1–3 μM) or L-Histidinol (HisOH, GCN2 activator; 4–16 mM), either alone or in combination for 24 h, compared to full medium. Mean ± SD of 3 technical replicates from 1 experiment (A), or mean ± SEM of 3 independent experiments (B). Data are expressed as fold change vs. control (full medium = 1). **P<0.01, ***P<0.001 (one way ANOVA, followed by Dunnett’s post-test vs. full medium). (PDF) [file pone.0200783.s005.pdf]
