# Supplementary material for: Amino acid deprivation triggers a novel GCN2-independent response leading to the transcriptional reactivation of non-native DNA sequences
Source: PLoS One. 2018 Jul 18;13(7):e0200783. doi: 10.1371/journal.pone.0200783 (PMC6051655; doi:10.1371/journal.pone.0200783)
Supplement: S6 Fig — (A) PCR amplification of GCN2 exon 1 and 6 from HeLa-OA1 parental cells and GCN2-KO clones 183#11 and 185#5 (targeted at exon 1), and 239 #1 (targeted at exon 6). The 185#5 clone contains multiple alleles, suggestive of a mixed cell population. (B) Sequence of GCN2 exon 1 in HeLa-OA1 parental cells (wild type) and GCN2-KO clone 183#11, showing a single base deletion. (C) Semi-quantitative PCR obtained by simultaneously amplifying GCN2 exon 1 (and exon 6 as normalization control) from HeLa-OA1 parental cells and GCN2-KO clone 183#11. The clone shows a significant amplification delay of exon 1, consistent with a deletion. Relative band density is shown below. (D) Sequence of GCN2 exon 6 in HeLa-OA1 parental cells (wild type) and GCN2-KO clone 239#1, showing a single base insertion and a 48 bp deletion in the clone. The original guide sequence is shown in bold red. (PDF) [file pone.0200783.s006.pdf]

S6 Fig

A

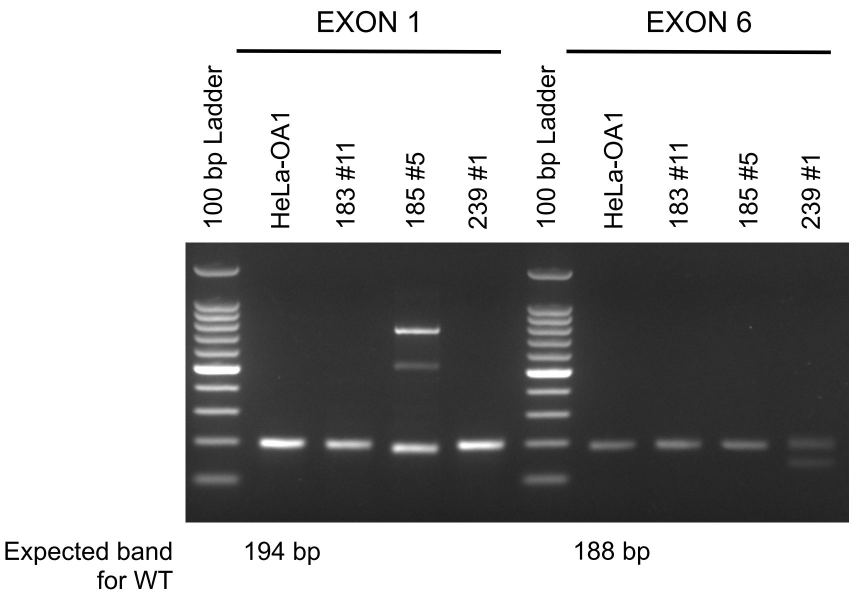

B

EXON 1

HeLa-OA1 (WT) CCATTTACGG**CGCGGACTTCCAAGACCTGCGG**CCGGACGCTT

183 #11 CCATTTACGG**CGCGGACTTCCAAGAC-TGCGG**CCGGACGCTT Allele 1 → single base deletion

C

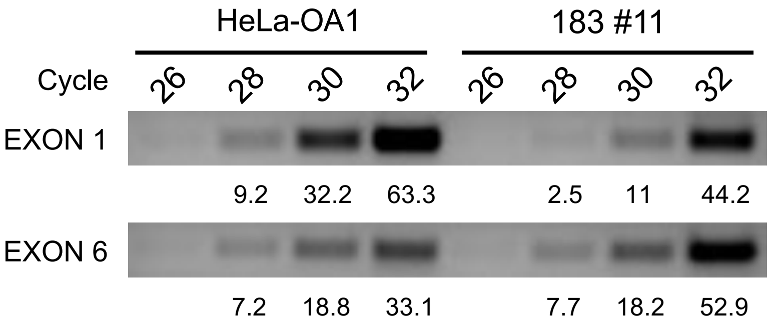

Allele 2 → probable deletion

D

EXON 6

HeLa-OA1 (WT) GGAGGACACA**GAACGGCTGCCATTCTACATGG**AGGCTCTCCT

239 #1 GGAGGACACA**GAACGGCTGCCATTCTTACATGG**AGGCTCTCCT Allele 1 → single base insertion

239 #1 **---Δ48bp--CATGG**AGGCTCTCCT Allele 2 → 48 bp deletion
