# Supplementary material for: Amino acid deprivation triggers a novel GCN2-independent response leading to the transcriptional reactivation of non-native DNA sequences
Source: PLoS One. 2018 Jul 18;13(7):e0200783. doi: 10.1371/journal.pone.0200783 (PMC6051655; doi:10.1371/journal.pone.0200783)
Supplement: S7 Fig — (A) PCR amplification of GCN2 exon 1 from HepG2-OA1 parental cells and GCN2-KO clone F27 (targeted at exon 1). (B) Sequence of GCN2 exon 1 in HepG2-OA1 parental cells (wild type) and GCN2-KO clone F27, showing a two bp insertion for allele 1 and an 18 bp deletion for allele 2. The original guide sequence is shown in bold red. (PDF) [file pone.0200783.s007.pdf]

S7 Fig

A

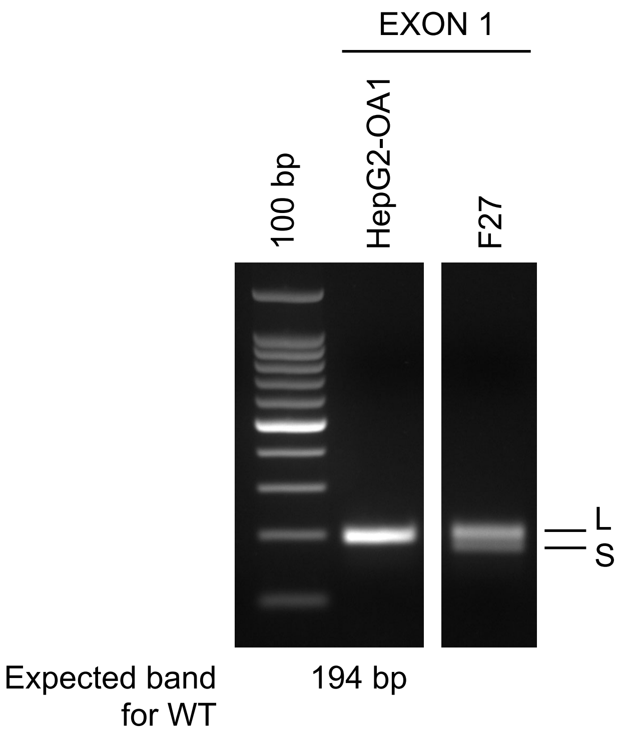

B

EXON 1

HepG2-OA1 (WT)	GACTTCCAAG**ACCTGCGGCCGGACGCTTGCGG**ACCGGTAGGA	
F27 L	GACTTCCAAG**ACCTGCGGCCGGACGCAATTGCGG**ACCGGTAGGA	Allele 1 → two bp insertion
F27 S	GACTTCC**-------------Δ18 bp--------------------TTTGCGG**ACCGGTAGGA	Allele 2 → 18 bp deletion
